# Polycystic Kidney Disease-Related Mortality in the US (1999-2024): A Nationwide Joinpoint Analysis of Trends, Disparities, and Comorbidity Structure

**DOI:** 10.7759/cureus.114792

**Published:** 2026-08-19

**Authors:** Hamza B Amir, Muhammad Uzair, Zenab M Khan, Haashir A Siddiqi, Astad Y Sidhwa

**Affiliations:** 1 Internal Medicine, Independent Research, Karachi, PAK

**Keywords:** age-adjusted mortality rate, autosomal dominant polycystic kidney disease, cdc wonder, health disparities, icd-10 q61.3, joinpoint regression, mortality trends, multiple cause of death, polycystic kidney disease

## Abstract

Introduction

Polycystic kidney disease (PKD) is the most prevalent inherited kidney disorder and a major cause of kidney failure, yet national mortality data specific to it remain scarce. No study has characterized US PKD-related mortality trends across the full period of the International Classification of Diseases, Tenth Revision (ICD-10) mortality coding.

Methods

We conducted a serial cross-sectional analysis of death certificate data from the Centers for Disease Control and Prevention Wide-ranging Online Data for Epidemiologic Research (CDC WONDER) Multiple Cause of Death database (bridged-race file 1999-2020; single-race file 2021-2024) among US adults aged 25 years and older. PKD-related deaths were identified by ICD-10 codes Q61.2 or Q61.3 recorded anywhere on the certificate. Age-adjusted mortality rates (AAMRs) per 100,000 (2000 US standard population) were stratified by sex, race and Hispanic origin, census region, age, urbanization, state, and place of death. Temporal trends were assessed with the Joinpoint Regression Program version 6.0.1 (grid search, 0-4 joinpoints; Monte Carlo permutation test, 4,499 permutations; log-linear model), reporting annual percent change (APC) and average APC (AAPC) with 95% CIs. Five hypotheses were specified before analysis.

Results

Between 1999 and 2024, 17,141 PKD-related deaths were recorded. The overall AAMR was 0.341 per 100,000 in 1999, fell to a nadir of 0.239 in 2013, and recovered to 0.311 by 2024. Joinpoint regression identified a single inflection in 2013: a 14-year decline (APC -3.01%; 95% CI, -4.00 to -2.01; p < 0.001) followed by an 11-year rise (APC +2.34%; 95% CI, +0.90 to +3.80; p = 0.003). The AAPC of -0.69% (p = 0.089) masked this two-segment trajectory. The reversal appeared across nearly all strata, with joinpoints clustered between 2008 and 2015, and was steepest among non-Hispanic Black decedents (segment-2 APC +4.17%; p = 0.010), approximately twice the non-Hispanic White rate (+2.07%). The Northeast was the only region without a significant rebound. Renal failure co-mention declined (AAPC -2.40%) while hypertension co-mention rose (AAPC +3.92%); ischemic heart disease co-mention declined (AAPC -2.45%). The underlying cause fell within the ICD-10 chapter containing PKD in 53.3% of classified deaths.

Conclusions

US PKD-related mortality reversed direction in 2013 after 14 years of decline. The reversal preceded the approval of ADPKD-specific therapy; its timing did not support a temporal association with the 2014 revision of kidney allocation policy. The rebound was numerically steepest among non-Hispanic Black decedents, and population-based co-mention rates shifted from renal failure toward hypertension.

## Introduction

Autosomal dominant polycystic kidney disease (ADPKD) is the most common inherited kidney disorder, arising predominantly from mutations in PKD1 or PKD2 and producing a progressive, multisystem disease with renal and extrarenal manifestations [1]. It is the leading heritable cause of kidney failure [2]. Although common relative to other inherited kidney diseases, ADPKD remains rare and frequently underdiagnosed: diagnosed prevalence in the US was estimated at 4.3 per 10,000 in national ambulatory care data, corresponding to roughly 140,000 diagnosed individuals [3], while a meta-analysis of European data estimated an average prevalence near 2.7 per 10,000 [4]. Because death certificate data identify deaths related to polycystic kidney disease (PKD) rather than a genetically confirmed diagnosis, such deaths among adults are understood to reflect ADPKD predominantly, given that the recessive form rarely survives to adulthood. The disease accounts for an estimated 8-10% of end-stage renal disease in the US and Europe [5].

Cardiovascular disease is the major cause of mortality in ADPKD, and hypertension, which appears early and in a large proportion of patients before any decline in kidney function, is its most common and most modifiable manifestation [6]. The comorbidity structure of these deaths is not fixed. In a Danish cohort of patients with ADPKD and end-stage renal disease, cardiovascular and cerebrovascular deaths declined between 1993 and 2008 [7]. In April 2018, tolvaptan became the first disease-specific therapy approved in the US to slow kidney function decline in adults at risk of rapidly progressing ADPKD, on the basis of the TEMPO 3:4 and REPRISE trials [8,9], and deceased-donor kidney allocation was restructured with the revised Kidney Allocation System implemented in December 2014 [10]. Joinpoint regression of death certificate data from the Centers for Disease Control and Prevention Wide-ranging Online Data for Epidemiologic Research (CDC WONDER) database has become an established approach for characterizing national trends in kidney-related mortality [11].

Despite this, mortality data specific to ADPKD are limited. The most recent US estimate relied on the US Renal Data System and was confined to the treated chronic kidney disease and end-stage renal disease population over a three-year window (2014-2016), providing point estimates rather than long-term trends [2]. Evidence on how the causes of death in ADPKD change over time derives from a non-US, registry-based cohort restricted to patients with end-stage renal disease and ending in 2008, before disease-specific therapy became available [7]. No study has examined national PKD-related mortality across the full period of the International Classification of Diseases, Tenth Revision (ICD-10) mortality coding, and none has characterized its trajectory through the years spanning the approval of tolvaptan and the revision of kidney allocation policy. Whether national PKD-related mortality has risen, fallen, or reversed direction has not been established, nor whether any such change differs by sex, race and Hispanic origin, or census region. Whether the comorbidity and underlying-cause structure of these deaths has shifted at the national level is likewise unknown. Prior CDC WONDER analyses of the ICD-10 Q61 code group examined it only within broader congenital-anomaly groupings restricted to pediatric ages, leaving adult PKD-related mortality unexamined [12].

We therefore conducted a nationwide, serial cross-sectional analysis of PKD-related mortality among US adults aged 25 years and older from 1999 to 2024, using CDC WONDER multiple-cause-of-death data and joinpoint regression. The primary aim was to characterize national trends in age-adjusted PKD-related mortality, identifying the timing and magnitude of any changes in trend. Secondary aims were to quantify trends across sex, race and Hispanic origin, and US census region; to describe the demographic, age, geographic, urbanization, state-level, and place-of-death distribution of decedents; to characterize the comorbidity co-mention profile and its temporal trends; and to describe the underlying-cause composition of PKD-related deaths. Because joinpoint regression identifies when and by how much a trend changed but not why, we further specified, before analysis, whether any observed change in trend coincided temporally with the April 2018 approval of tolvaptan or the December 2014 revision of the Kidney Allocation System and whether trends differed across demographic strata. To our knowledge, this is the first national analysis of PKD-related mortality trends in US adults.

## Materials and methods

Study design and data source

We conducted a serial cross-sectional analysis of national mortality among US adults using death certificate data from the CDC WONDER Multiple Cause of Death database [13]. Cases were ascertained on a multiple-cause basis: a death was included when a qualifying PKD code appeared in the multiple-cause-of-death fields of the certificate, whether recorded as the underlying cause or as a contributing cause. The study period was 1999 through 2024.

Two CDC WONDER Multiple Cause of Death files were combined to construct a continuous series spanning a change in the racial classification of the underlying mortality files. The 1999-2020 file, which uses bridged-race population estimates, supplied all years from 1999 through 2020, inclusive of 2018, 2019, and 2020. The expanded 2018-2024 file, which uses single-race population estimates, supplied only the years 2021 through 2024. No calendar year was drawn from more than one file, and the overlapping 2018-2020 window available in the expanded file was not used. Bridged-race population estimates redistribute persons reporting more than one race into the four single-race categories used before the 2000 Census standard, providing consistent denominators for 1999-2020; single-race estimates, used for the 2021-2024 data, retain the multiple-race categories collected since the 2000 Census without such bridging. Because the two schemes are not directly interchangeable, the files were combined without overlap, and race was collapsed to categories defined consistently across both. Data were accessed on June 15, 2026, and all analyses were conducted on June 18, 2026.

Case definition

Cases comprised deaths assigned ICD-10 [14] code Q61.2 (polycystic kidney, adult type) or Q61.3 (polycystic kidney, unspecified) in any multiple-cause field. We excluded Q61.1 (autosomal recessive PKD, a clinically distinct entity) and the remaining Q61 subcodes Q61.0, Q61.4, Q61.5, Q61.8, and Q61.9.

Inclusion of Q61.3 alongside Q61.2 was an ascertainment decision defined before analysis, on two grounds. First, the four-character WHO ICD-10 used by CDC WONDER provides no adult-specific subcode beyond Q61.2, so no more granular code is available to certifiers who wish to designate adult-type disease. Second, the adult-type designation is recorded inconsistently on death certificates, and restriction to Q61.2 alone would omit most PKD deaths. Because the unspecified code cannot on its own distinguish the dominant from the recessive form, the cohort is described throughout as PKD-related mortality. Autosomal dominant disease is not asserted as the ascertained diagnosis. Among adults, deaths in this code group are expected to reflect ADPKD predominantly, because the recessive form infrequently survives to adulthood, and the age restriction described below is the operational basis for that expectation.

Study population and age restriction

Analyses were restricted to adults aged 25 years and older, a threshold defined before analysis. The rationale is that the unspecified code Q61.3 captures a component of recessive and infantile disease concentrated at the extremes of early life, and restriction to ages 25 and above removes that early-life component while retaining the adult cohort of interest. Age was operationalized using the CDC WONDER ten-year age groups from 25-34 years through 85 years and older, and identical age groups were used in both source files.

Outcome measure and rate construction

The outcome was the age-adjusted mortality rate (AAMR) per 100,000 population, computed by CDC WONDER using the direct method with the 2000 US standard population [15]. CDC WONDER reports each age-adjusted rate with its lower and upper 95% confidence limits and does not report a standard error for the age-adjusted rate. The standard error required for regression weighting was therefore derived from the reported confidence limits for each rate as the interval width divided by 3.92, that is, (upper limit - lower limit) / 3.92. Derived standard errors were supplied to the regression program, which fitted each series with an uncorrelated-error specification using the provided standard errors, producing a weighted fit rather than assuming constant variance. This derivation assumes that the reported confidence limits are approximately symmetric about the point estimate. For the age-adjusted rates modeled here, all of which were based on 20 or more deaths per year, the CDC WONDER intervals were close to symmetric, so the resulting standard errors are a reasonable approximation.

CDC WONDER suppresses death counts below 10 and flags rates computed from fewer than 20 deaths as unreliable. Every stratum carried forward to regression modeling was based on 20 or more deaths in each analytic year, with no suppressed and no flagged year in any modeled series. Strata that did not meet this count in a sufficient number of years were summarized descriptively rather than modeled, as specified in the following section.

Stratification

National trends were examined overall and within prespecified strata. Sex was analyzed as male and female. Race and ethnicity were collapsed into three groups: non-Hispanic White, non-Hispanic Black, and Hispanic. Collapsing to three broad groups was necessary because the two source files classify race under different schemes, bridged race through 2020 and single race from 2021, and these three categories are defined consistently across both schemes, which preserves comparability across the file boundary. Geographic analyses used the four US census regions of Northeast, Midwest, South, and West.

The remaining race and ethnicity groups, comprising non-Hispanic Asian or Pacific Islander, non-Hispanic American Indian or Alaska Native, non-Hispanic Native Hawaiian or Other Pacific Islander, and persons of more than one race, did not sustain the death counts required for stable joinpoint estimation across the study period and were tabulated descriptively rather than modeled. Records with unknown or unstated Hispanic origin were retained in the overall analysis and excluded only from the race- and ethnicity-specific strata.

Comorbidity co-mention definitions

A comorbidity was counted as co-mentioned when its ICD-10 code appeared in the multiple-cause fields of a certificate that also carried a qualifying PKD code. Co-mention denotes co-listing on the same certificate. It does not establish that the comorbidity caused death, and these deaths are described throughout as having a condition co-listed rather than as deaths from that condition.

Three co-mention series were modeled as age-adjusted rates by joinpoint regression: renal failure (N17-N19), hypertension (I10-I15), and ischemic heart disease (I20-I25). The hypertension range I10-I15 comprises essential, hypertensive-renal, and hypertensive-heart categories, and no attempt was made to separate these. A further panel of co-mentioned conditions was characterized descriptively over the full period, comprising heart failure (I50), cerebrovascular disease (I60-I69), subarachnoid hemorrhage (I60), sepsis (A40-A41), and cystic liver disease (Q44.6). Subarachnoid hemorrhage was queried separately as a nested subset of the cerebrovascular category rather than as a mutually exclusive group, so the two overlap, and it was too infrequent to support a joinpoint model. The proportion of PKD deaths carrying each comorbidity was reported descriptively, without inferential trend testing.

Underlying-cause composition

The distribution of the underlying cause of death across ICD-10 chapters was tabulated as a proportional composition and reported descriptively. No inferential trend testing was applied to this distribution.

Statistical analysis

Temporal trends were modeled with the National Cancer Institute Joinpoint Regression Program, version 6.0.1 [16]. Age-adjusted rates were log-transformed so that each fitted segment corresponds to a constant annual percent change (APC). The multiplicative form is the standard model for mortality-rate trends. Models were fitted by the grid search method, permitting zero to four joinpoints, with a minimum of two observations between a joinpoint and either end of the series and a minimum of two observations between adjacent joinpoints. The number of joinpoints was selected by a Monte Carlo permutation test with 4,499 permutations at an overall significance level of 0.05 [16]. CIs for the APC of each segment and for the average APC (AAPC) across the full range were computed by the parametric method. Errors were treated as uncorrelated, with heteroscedasticity accommodated through the supplied standard errors. For each series, we report the APC and its CI for every segment, the identified joinpoint years, and the AAPC for 1999-2024. All tests were two-sided at α = 0.05. Although the grid-search procedure permitted up to four joinpoints, the permutation test constrains model complexity by requiring each additional joinpoint to be statistically justified; in practice, the selected models were parsimonious, with the overall series and most strata resolving to a single joinpoint and only the hypertension series to two, so the four-joinpoint ceiling did not drive the results. The overall, sex, and race- and ethnicity-specific analyses were prespecified primary and secondary analyses; the region- and comorbidity-specific analyses, and any segment with a borderline p-value, are regarded as exploratory in view of the number of trends examined and the absence of formal multiplicity adjustment.

The AAPC and the segment APCs answer different questions, and this distinction governed reporting. Where the segment APCs within a series were statistically significant but opposite in sign, a nonsignificant full-range AAPC reflects the arithmetic cancellation of opposing segment trends and is not evidence that the rate was unchanging. In such series, interpretation was led by the segment-specific APCs and the joinpoint location, and the AAPC was reported as the net change across the full period rather than as a summary of the trend.

Prespecified hypotheses

Five hypotheses were specified before analysis. Joinpoint regression identifies when a trend changed and the magnitude of the change. It does not identify the cause. Each hypothesis was therefore framed as a test of temporal coincidence between an external event and an observed joinpoint, not as a test of causation. The hypotheses were as follows: that a change in trend would coincide with the US approval of tolvaptan, the first disease-specific therapy for ADPKD, in April 2018 [8,9]; that a change in trend would coincide with the 2014 revision of the Kidney Allocation System; that trends would differ across race and ethnicity groups; that co-mentioned subarachnoid hemorrhage would decline across the period; and that the comorbidity composition of PKD deaths would change across the period. Specifying these relationships in advance allows a hypothesis unsupported by the data to be reported as such, which constrains interpretation more credibly than a relationship identified after inspecting the results.

Sensitivity analysis

To quantify the influence of the ascertainment decision, the case definition was repeated using Q61.2 alone, and the resulting cohort was compared with the primary Q61.2-plus-Q61.3 cohort. This analysis measures the contribution of the unspecified code to case capture and indicates whether the primary cohort depends materially on its inclusion. To assess whether the transition between the bridged-race and single-race population files affected the estimated rates, we compared age-adjusted rates for the three overlapping years (2018-2020) available in both files, which quantifies any discontinuity attributable to the change in population-estimation scheme.

Multiple comparisons

Thirteen prespecified joinpoint analyses were performed. Given this multiplicity, segment APCs with p-values near 0.05 warrant cautious interpretation. No formal alpha adjustment was applied, as Bonferroni correction is overly conservative for segmented models with non-independent segments. Interpretation prioritized the magnitude and consistency of trends over nominal significance.

Descriptive supplements

The age distribution of the cohort, the distribution of place of death, and state-level mortality were summarized descriptively. Analysis by urbanization was restricted to 1999-2020 because the expanded 2018-2024 file does not include age-adjusted rates stratified by urbanization. This is a constraint of the data source rather than an analytic choice.

Ethics and reporting

This study used de-identified, publicly available aggregate data and does not constitute human-subjects research. Institutional review board approval was therefore not required. The analysis is reported in accordance with the REporting of studies Conducted using Observational Routinely-collected health Data (RECORD) statement, an extension of the STrengthening the Reporting of OBservational studies in Epidemiology (STROBE) guideline for studies using routinely collected health data [17]. All data are publicly available through CDC WONDER. Joinpoint regression was performed with the Joinpoint Regression Program version 6.0.1. Descriptive tabulation and figure production used Python.

## Results

Overall mortality burden and descriptive distribution

Between 1999 and 2024, 17,141 PKD-related deaths (ICD-10 Q61.2 or Q61.3, listed as any cause of death) were recorded among US adults aged 25 years and older. Of these, 13,780 occurred during 1999-2020 (bridged-race file) and 3,361 during 2021-2024 (single-race file). Annual death counts rose from 643 in 1999 to 825 in 2024, reaching a low of 539 in 2008 and a high of 903 in 2022. The overall AAMR was 0.341 per 100,000 in 1999, declined to a nadir of 0.239 in 2013, and recovered to 0.311 by 2024. The demographic, geographic, and urbanization distribution of decedents is summarized in Table 1.

**Table 1:** Demographic, geographic, and urbanization distribution of PKD-related deaths (ICD-10 Q61.2 and Q61.3) in the US, 1999-2024. Deaths are counts of records with ICD-10 code Q61.2 or Q61.3 listed as any cause of death among adults aged 25 years and older, accessed via the CDC WONDER Multiple Cause of Death files (bridged-race file 1999-2020; single-race file 2021-2024). Unless otherwise stated, percentages are of the overall 17,141 deaths. The crude rate per 100,000 population-years was computed as total deaths divided by summed annual population, with 95% CIs by normal approximation. AAMR values are observed age-adjusted rates for 1999 and 2024 taken from each stratum’s joinpoint series, standardized to the 2000 US standard population by the direct method. Age-group deaths sum to 17,128; the 13-death shortfall reflects CDC WONDER suppression of cells containing fewer than 10 deaths. Urbanization was analyzed for 1999-2020 only, because the 2018-2024 file does not include age-adjusted rates stratified by urbanization; urbanization percentages are therefore of the 13,780 deaths occurring in that period. Place-of-death categories sum to 16,997; the 144-death shortfall reflects suppressed and unclassified records, and place of death is reported as counts only because no population denominator applies to that stratification. AAMR, age-adjusted mortality rate; CDC WONDER, Centers for Disease Control and Prevention Wide-ranging Online Data for Epidemiologic Research; ICD-10, International Classification of Diseases, Tenth Revision; NCHS, National Center for Health Statistics; PKD, polycystic kidney disease

Characteristic	Deaths, N (%)	Population-years	Crude rate per 100,000 (95% CI)	AAMR 1999 (per 100,000)	AAMR 2024 (per 100,000)
Overall					
Overall (US, adults ≥25 years)	17,141 (100.0)	5,398,746,349	0.317 (0.313-0.322)	0.341	0.311
Sex					
Male	9,485 (55.3)	2,605,943,716	0.364 (0.357-0.371)	0.441	0.395
Female	7,656 (44.7)	2,792,802,633	0.274 (0.268-0.280)	0.309	0.236
Race and Hispanic origin					
Non-Hispanic White	13,201 (77.0)	3,673,762,428	0.359 (0.353-0.365)	0.353	0.318
Non-Hispanic Black	2,160 (12.6)	630,793,625	0.342 (0.328-0.357)	0.415	0.342
Hispanic or Latino	1,139 (6.6)	742,520,272	0.153 (0.144-0.162)	0.456	0.178
Age group (10-year bands)					
25-34 years	333 (1.9)	1,103,082,254	0.030 (0.027-0.033)	-	-
35-44 years	986 (5.8)	1,108,316,424	0.089 (0.083-0.095)	-	-
45-54 years	2,258 (13.2)	1,089,970,438	0.207 (0.199-0.216)	-	-
55-64 years	3,679 (21.5)	934,829,484	0.394 (0.381-0.406)	-	-
65-74 years	4,191 (24.5)	648,043,148	0.647 (0.627-0.666)	-	-
75-84 years	3,737 (21.8)	369,898,963	1.010 (0.978-1.043)	-	-
85+ years	1,944 (11.3)	144,605,638	1.344 (1.285-1.404)	-	-
US Census region					
Northeast	2,869 (16.7)	989,259,418	0.290 (0.279-0.301)	0.302	0.278
Midwest	4,146 (24.2)	1,158,892,622	0.358 (0.347-0.369)	0.383	0.318
South	5,583 (32.6)	2,007,516,980	0.278 (0.271-0.285)	0.313	0.259
West	4,543 (26.5)	1,243,077,329	0.365 (0.355-0.376)	0.479	0.357
2013 NCHS urbanization classification (1999-2020; % of 13,780)					
Large Central Metro	3,843 (27.9)	1,364,782,777	0.282 (0.273-0.290)	-	-
Large Fringe Metro	2,812 (20.4)	1,107,354,420	0.254 (0.245-0.263)	-	-
Medium Metro	3,088 (22.4)	918,036,376	0.336 (0.325-0.348)	-	-
Small Metro	1,515 (11.0)	405,040,249	0.374 (0.355-0.393)	-	-
Micropolitan (Nonmetro)	1,445 (10.5)	392,223,879	0.368 (0.349-0.387)	-	-
NonCore (Nonmetro)	1,077 (7.8)	286,410,290	0.376 (0.354-0.398)	-	-
Place of death					
Medical facility - inpatient	6,755 (39.4)	-	-	-	-
Decedent’s home	5,352 (31.2)	-	-	-	-
Nursing home/long-term care	1,755 (10.2)	-	-	-	-
Medical facility - outpatient or ER	1,309 (7.6)	-	-	-	-
Hospice facility	1,026 (6.0)	-	-	-	-
Other	776 (4.5)	-	-	-	-
Medical facility - dead on arrival	24 (0.1)	-	-	-	-

Mortality was male-predominant: 9,485 deaths (55.3%) occurred among men and 7,656 (44.7%) among women, with pooled crude rates of 0.364 and 0.274 per 100,000 population-years, respectively. By race and Hispanic origin, non-Hispanic White decedents accounted for 13,201 deaths (77.0%), non-Hispanic Black for 2,160 (12.6%), and Hispanic or Latino for 1,139 (6.6%). Deaths rose steeply with age, the crude rate increasing monotonically from 0.030 per 100,000 in the 25- to 34-year stratum to 1.344 in the 85-and-older stratum, and the largest single age band was 65-74 years (4,191 deaths, 24.5% of the cohort). Age-classified deaths summed to 17,128, and the 13-death difference from the overall total reflects suppression of age-by-year cells with fewer than 10 deaths. By US census region, the South contributed the largest absolute burden (5,583 deaths, 32.6%), followed by the West (4,543, 26.5%), Midwest (4,146, 24.2%), and Northeast (2,869, 16.7%). Analysis by 2013 National Center for Health Statistics urbanization classification, available for 1999-2020, showed the largest share in large central metropolitan counties (3,843, 27.9% of the 13,780 deaths in that period). By place of death, most deaths occurred as a medical-facility inpatient (6,755, 39.4%) or at the decedent’s home (5,352, 31.2%); place-of-death categories summed to 16,997, the 144-death shortfall reflecting suppressed and unclassified records.

Overall temporal trend in age-adjusted mortality

**Figure 1:**
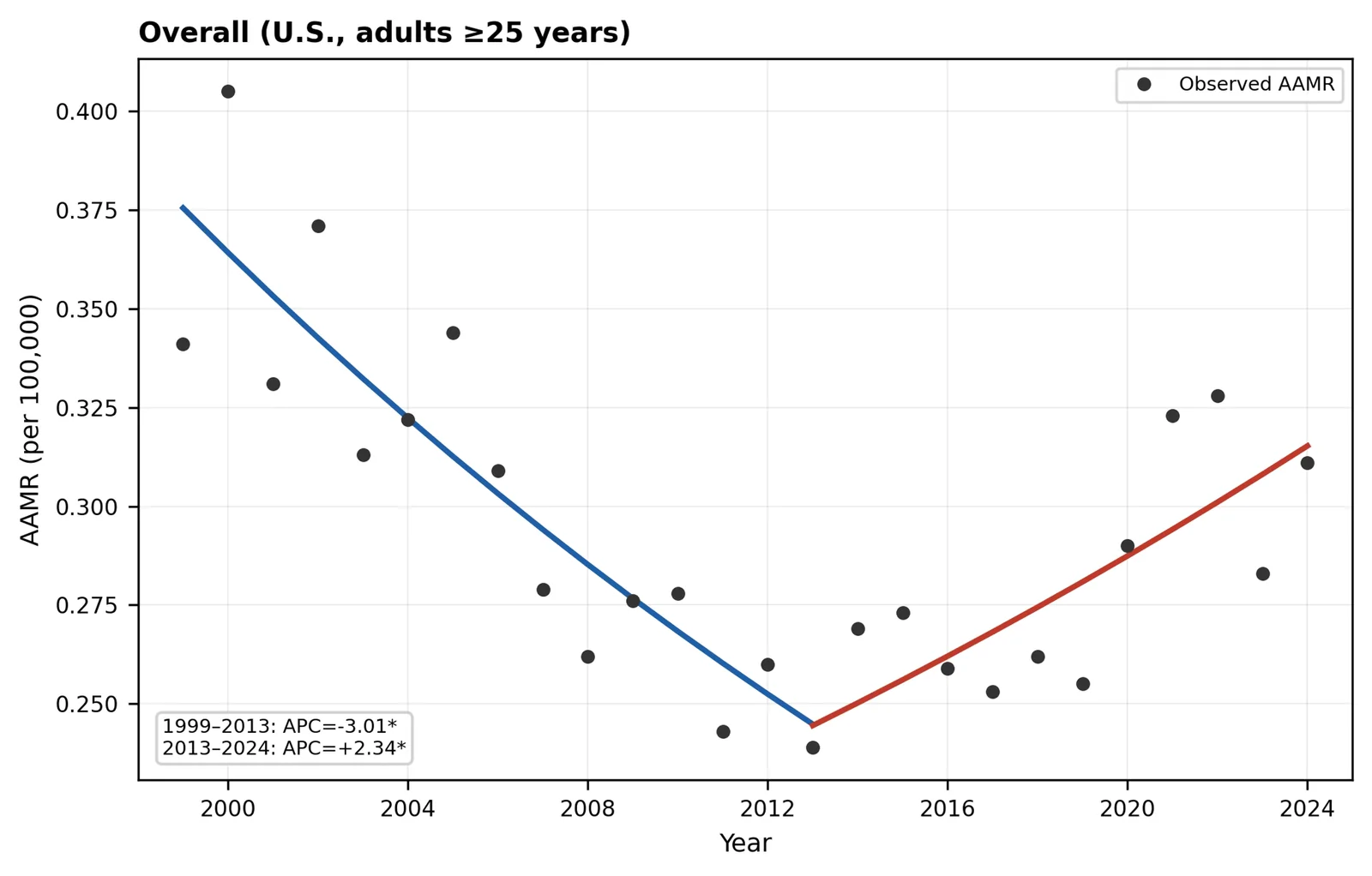
AAMRs for PKD-related deaths (ICD-10 Q61.2 and Q61.3), US adults aged 25 years and older, 1999-2024. * APC is significantly different from zero at α = 0.05. Filled circles show observed annual AAMRs per 100,000, standardized to the 2000 US standard population. Solid segments show the fitted joinpoint regression (NCI Joinpoint Regression Program version 6.0.1; log-linear model; uncorrelated errors; grid search; Monte Carlo permutation test with 4,499 permutations; α = 0.05). Fitted values were computed as exp(β₀ + β₁ × year) using segment-specific parameters from the Joinpoint model estimates. Parametric 95% CIs are reported for all APC and AAPC estimates. A single joinpoint was identified in 2013 (95% CI, 2010-2016). Segment 1 (1999-2013): APC -3.01% (95% CI, -4.00 to -2.01), p < 0.001. Segment 2 (2013-2024): APC +2.34% (95% CI, +0.90 to +3.80), p = 0.003. AAPC over 1999-2024: -0.69% (95% CI, -1.48 to +0.11), p = 0.089. The nonsignificant AAPC reflects the offset between the declining and rising segments rather than a stable underlying rate. Line color denotes successive trend segments defined by the joinpoint(s): blue, the first segment; red, the second segment. AAMR, age-adjusted mortality rate; APC, annual percent change; AAPC, average annual percent change; PKD, polycystic kidney disease

Joinpoint regression of the overall AAMR identified a single joinpoint in 2013, partitioning the 26-year series into two statistically distinct segments (Figure 1, Table 2). The rate declined at an APC of -3.01% (95% CI, -4.00 to -2.01; p < 0.001) from 1999 to 2013, then rose at +2.34% per year (95% CI, +0.90 to +3.80; p = 0.003) from 2013 to 2024. The AAPC across the full 1999-2024 period was -0.69% (95% CI, -1.48 to +0.11; p = 0.089) and was not statistically significant. Because the two segments were individually significant and opposite in sign, this nonsignificant AAPC reflects the cancellation of a declining and a rising segment rather than a stable underlying rate. Throughout the analysis, series with opposing significant segments are interpreted by their segment-specific APCs and joinpoint location, with the AAPC reported as the net change across the period. Full annual observed and fitted rates with standard errors, final model parameters, and the Monte Carlo permutation-test sequences for every stratum are provided in Appendices A-D, respectively. Annual death counts for every analyzed stratum are tabulated in Appendix D.

**Table 2:** Joinpoint regression analysis of AAMRs for PKD-related deaths (ICD-10 Q61.2 and Q61.3) in the US, 1999-2024, by sex, race and Hispanic origin, US census region, and comorbidity co-mention. * APC or AAPC significantly different from zero at α = 0.05 (two-sided). AAMRs per 100,000, standardized to the 2000 US standard population by the direct method. Deaths, N (%) are of the overall 17,141 PKD-related deaths; for the three comorbidity series, N is the co-mention count and the percentage is of the same denominator. Joinpoint regression was fit using the NCI Joinpoint Regression Program version 6.0.1 with a log-linear model, uncorrelated errors, grid search, and Monte Carlo permutation test for model selection (4,499 permutations; overall significance level α = 0.05). The minimum number of joinpoints allowed was zero and the maximum was four; at least two observations were required between adjacent joinpoints and between any joinpoint and the series endpoints. Parametric 95% CIs were used for APC and AAPC. Segment 1 refers to the period before the first identified joinpoint and segment 2 to the period after; the PKD and hypertension series was fit with two joinpoints and therefore has three segments, shown together in the segment 2/3 column. AAMR, age-adjusted mortality rate; APC, annual percent change; AAPC, average annual percent change; PKD, polycystic kidney disease

Stratum	Deaths, N (%)	AAMR 1999	AAMR 2024	Joinpoint year(s)	Trend segment 1: years, APC % (95% CI)	Trend segment 2/3: years, APC % (95% CI)	AAPC 1999-2024, % (95% CI)
Overall (US, adults ≥25 years)	17,141 (100.0)	0.341	0.311	2013	1999-2013: -3.01* (-4.00 to -2.01)	2013-2024: 2.34* (0.90 to 3.80)	-0.69 (-1.48 to 0.11)
Male	9,485 (55.3)	0.441	0.395	2014	1999-2014: -3.20* (-4.35 to -2.03)	2014-2024: 3.68* (1.64 to 5.76)	-0.50 (-1.50 to 0.51)
Female	7,656 (44.7)	0.309	0.236	2014	1999-2014: -2.95* (-3.75 to -2.15)	2014-2024: 2.11* (0.63 to 3.62)	-0.96* (-1.67 to -0.24)
Non-Hispanic White	13,201 (77.0)	0.353	0.318	2012	1999-2012: -2.78* (-3.61 to -1.95)	2012-2024: 2.07* (1.07 to 3.07)	-0.48 (-1.09 to 0.12)
Non-Hispanic Black	2,160 (12.6)	0.415	0.342	2015	1999-2015: -3.14* (-4.45 to -1.82)	2015-2024: 4.17* (1.08 to 7.37)	-0.57 (-1.87 to 0.74)
Hispanic or Latino	1,139 (6.6)	0.456	0.178	2010	1999-2010: -9.00* (-12.83 to -5.01)	2010-2024: 1.64 (-1.01 to 4.36)	-3.19* (-5.35 to -0.98)
Northeast	2,869 (16.7)	0.302	0.278	2008	1999-2008: -4.63* (-6.87 to -2.33)	2008-2024: 0.93 (-0.06 to 1.93)	-1.11* (-2.09 to -0.11)
Midwest	4,146 (24.2)	0.383	0.318	2013	1999-2013: -2.49* (-3.78 to -1.19)	2013-2024: 2.41* (0.53 to 4.32)	-0.36 (-1.39 to 0.68)
South	5,583 (32.6)	0.313	0.259	2014	1999-2014: -3.25* (-4.47 to -2.01)	2014-2024: 3.73* (1.43 to 6.09)	-0.51 (-1.61 to 0.60)
West	4,543 (26.5)	0.479	0.357	2014	1999-2014: -3.27* (-4.27 to -2.27)	2014-2024: 2.92* (1.17 to 4.70)	-0.84 (-1.70 to 0.03)
PKD + renal failure	8,260 (48.2)	0.195	0.105	2014	1999-2014: -3.76* (-4.76 to -2.75)	2014-2024: -0.32 (-2.42 to 1.82)	-2.40* (-3.37 to -1.42)
PKD + hypertension	5,172 (30.2)	0.038	0.127	2002, 2013	1999-2002: 24.08 (-4.52 to 61.25)	2002-2013: -3.16* (-6.12 to -0.10); 2013-2024: 6.26* (3.89 to 8.68)	3.92* (0.52 to 7.44)
PKD + ischemic heart disease	3,795 (22.1)	0.095	0.05	2013	1999-2013: -5.68* (-7.32 to -4.02)	2013-2024: 1.82 (-0.86 to 4.57)	-2.45* (-3.85 to -1.04)

Trends by sex

**Figure 2:**
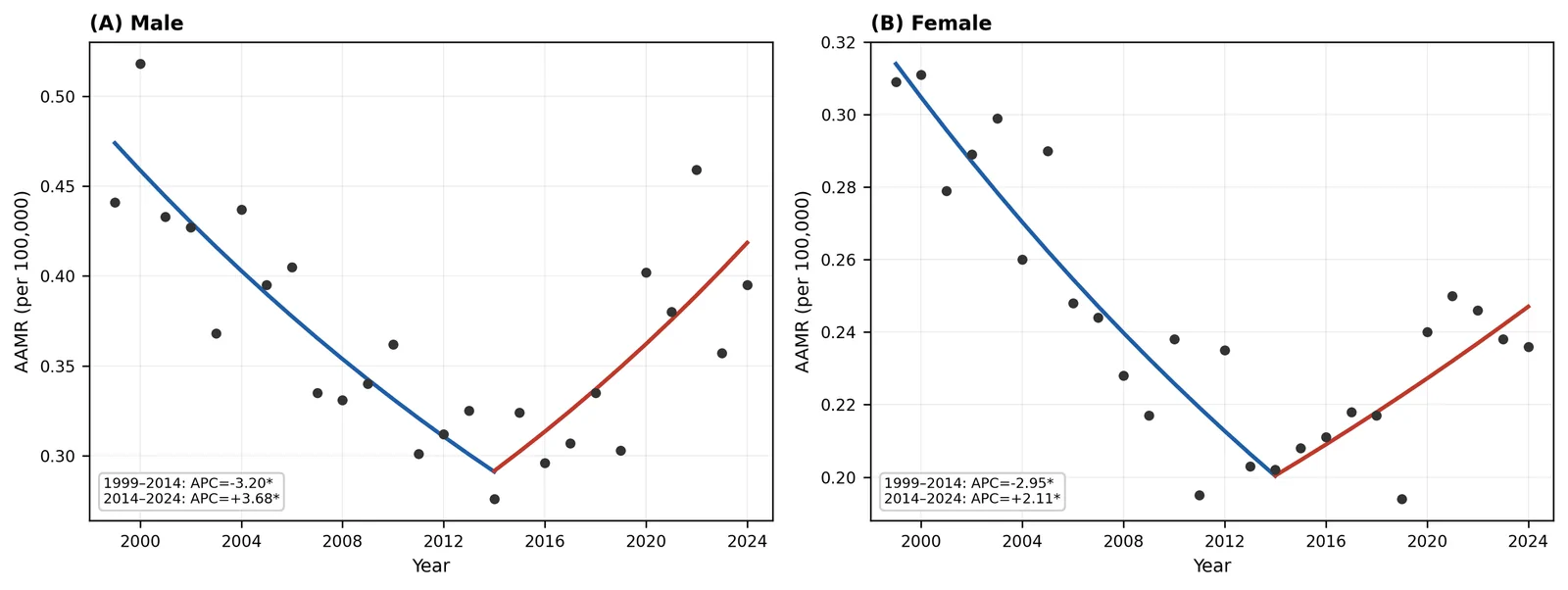
AAMRs for PKD-related deaths by sex, US, 1999-2024: (A) Male; (B) Female. * APC or AAPC significantly different from zero at α = 0.05. Filled circles show observed annual AAMRs per 100,000, standardized to the 2000 US standard population. Solid segments show the fitted joinpoint regression (NCI Joinpoint Regression Program version 6.0.1; log-linear model; uncorrelated errors; grid search; Monte Carlo permutation test with 4,499 permutations; α = 0.05). Fitted values were computed as exp(β₀ + β₁ × year) using segment-specific parameters from the Joinpoint model estimates. Parametric 95% CIs are reported for all APC and AAPC estimates. Both sexes inflected at 2014. Male: joinpoint 2014 (95% CI, 2011-2017); segment 1 (1999-2014) APC -3.20% (95% CI, -4.35 to -2.03), p < 0.001; segment 2 (2014-2024) APC +3.68% (95% CI, +1.64 to +5.76), p = 0.001; AAPC -0.50% (95% CI, -1.50 to +0.51), p = 0.328. Female: joinpoint 2014 (95% CI, 2010-2017); segment 1 APC -2.95% (95% CI, -3.75 to -2.15), p < 0.001; segment 2 APC +2.11% (95% CI, +0.63 to +3.62), p = 0.007; AAPC -0.96% (95% CI, -1.67 to -0.24), p = 0.009. The declining segments were closely matched between sexes, whereas the male rebound was steeper, producing a significant net decline in women only. Line color denotes successive trend segments defined by the joinpoint(s): blue, the first segment; red, the second segment. APC, annual percent change; AAPC, average annual percent change; PKD, polycystic kidney disease

Both sexes followed the two-segment pattern of decline followed by rebound (Figure 2, Table 2). Among men, a single joinpoint was identified at 2014. The AAMR declined at -3.20% per year (95% CI, -4.35 to -2.03; p < 0.001) from 1999 to 2014, then rose at +3.68% per year (95% CI, +1.64 to +5.76; p = 0.001) from 2014 to 2024, and the AAPC was -0.50% (95% CI, -1.50 to +0.51; p = 0.328), not significant. Among women, the joinpoint also fell at 2014, with a segment-1 APC of -2.95% (95% CI, -3.75 to -2.15; p < 0.001) and a segment-2 APC of +2.11% (95% CI, +0.63 to +3.62; p = 0.007). The female AAPC was -0.96% (95% CI, -1.67 to -0.24; p = 0.009) and was statistically significant.

Trends by race and Hispanic origin

**Figure 3:**
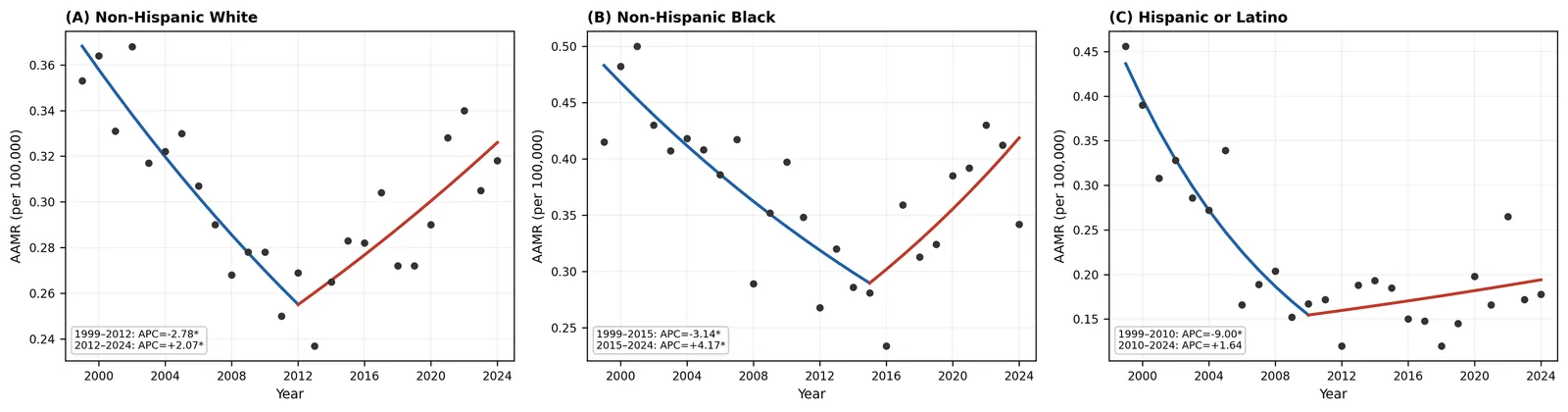
AAMRs for PKD-related deaths by race and Hispanic origin, US, 1999-2024: (A) Non-Hispanic White. (B) Non-Hispanic Black. (C) Hispanic or Latino. * APC or AAPC significantly different from zero at α = 0.05. Filled circles show observed annual AAMRs per 100,000, standardized to the 2000 US standard population. Solid segments show the fitted joinpoint regression (NCI Joinpoint Regression Program version 6.0.1; log-linear model; uncorrelated errors; grid search; Monte Carlo permutation test with 4,499 permutations; α = 0.05). Fitted values were computed as exp(β₀ + β₁ × year) using segment-specific parameters from the Joinpoint model estimates. Parametric 95% CIs are reported for all APC and AAPC estimates. Non-Hispanic White: joinpoint 2012 (95% CI, 2009-2014); segment 1 (1999-2012) APC -2.78% (95% CI, -3.61 to -1.95), p < 0.001; segment 2 (2012-2024) APC +2.07% (95% CI, +1.07 to +3.07), p < 0.001; AAPC -0.48% (95% CI, -1.09 to +0.12), p = 0.118. Non-Hispanic Black: joinpoint 2015 (95% CI, 2012-2017); segment 1 APC -3.14% (95% CI, -4.45 to -1.82), p < 0.001; segment 2 APC +4.17% (95% CI, +1.08 to +7.37), p = 0.010; AAPC -0.57% (95% CI, -1.87 to +0.74), p = 0.393. Hispanic or Latino: joinpoint 2010 (95% CI, 2006-2018); segment 1 (1999-2010) APC -9.00% (95% CI, -12.83 to -5.01), p < 0.001; segment 2 APC +1.64% (95% CI, -1.01 to +4.36), p = 0.214, not significant; AAPC -3.19% (95% CI, -5.35 to -0.98), p = 0.005. The second-segment rise was approximately twice as steep among non-Hispanic Black as among non-Hispanic White decedents. Line color denotes successive trend segments defined by the joinpoint(s): blue, the first segment; red, the second segment. AAMR, age-adjusted mortality rate; APC, annual percent change; AAPC, average annual percent change; PKD, polycystic kidney disease

Non-Hispanic White decedents showed a joinpoint at 2012, with a segment-1 decline of -2.78% per year (95% CI, -3.61 to -1.95; p < 0.001) and a segment-2 rise of +2.07% per year (95% CI, +1.07 to +3.07; p < 0.001). The AAPC was -0.48% (95% CI, -1.09 to +0.12; p = 0.118), not significant (Figure 3, Table 2). Non-Hispanic Black decedents showed a joinpoint at 2015, with a segment-1 APC of -3.14% (95% CI, -4.45 to -1.82; p < 0.001) and a segment-2 APC of +4.17% (95% CI, +1.08 to +7.37; p = 0.010). The AAPC was -0.57% (95% CI, -1.87 to +0.74; p = 0.393), not significant. For Hispanic or Latino decedents, a joinpoint was identified at 2010. The AAMR declined at -9.00% per year (95% CI, -12.83 to -5.01; p < 0.001) from 1999 to 2010, followed by a segment-2 change of +1.64% per year (95% CI, -1.01 to +4.36; p = 0.214) that was not statistically significant, and the Hispanic AAPC was -3.19% (95% CI, -5.35 to -0.98; p = 0.005) and was significant.

Trends by US census region

**Figure 4:**
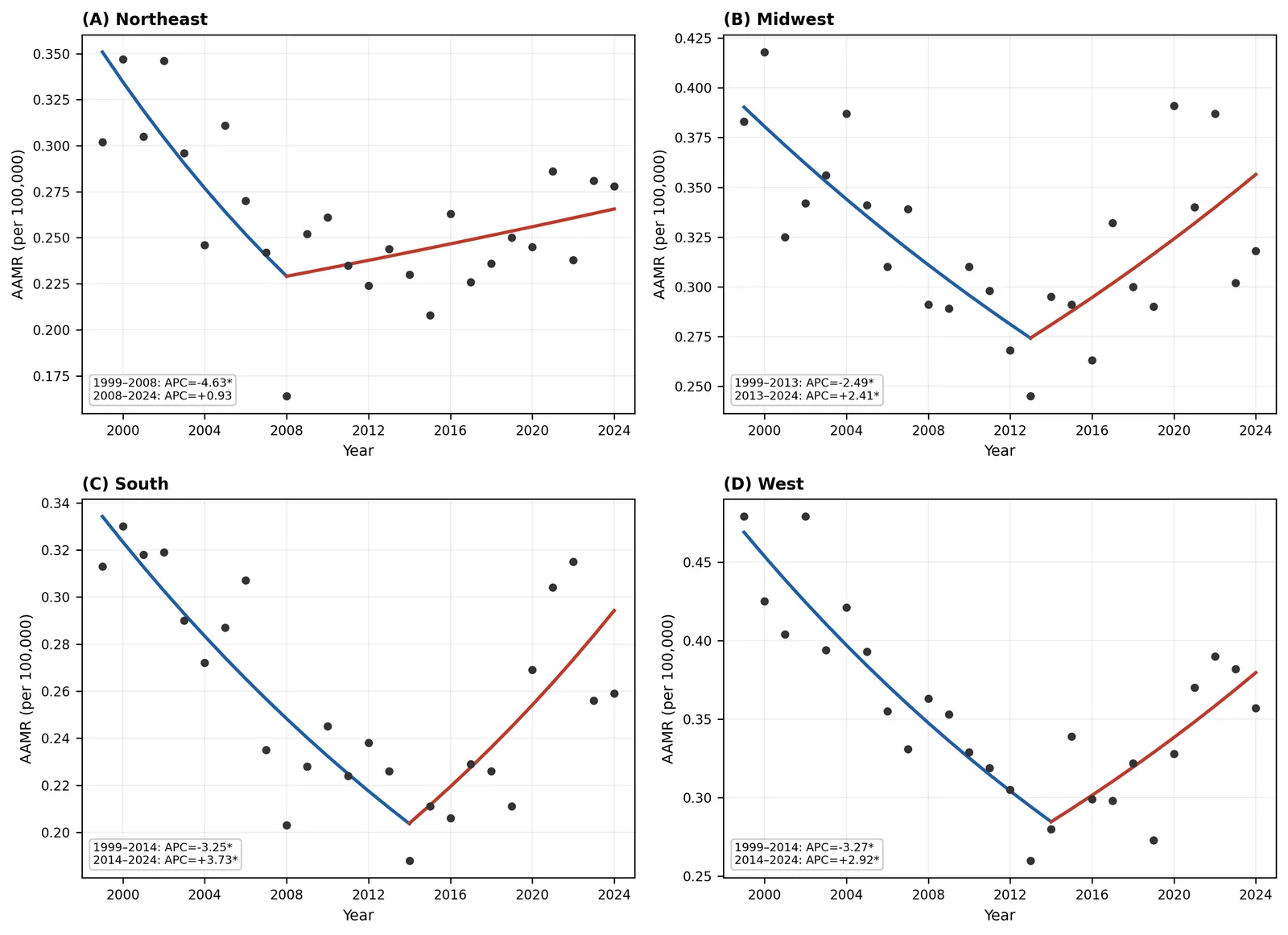
AAMRs for PKD-related deaths by US census region, 1999-2024: (A) Northeast. (B) Midwest. (C) South. (D) West. * APC or AAPC significantly different from zero at α = 0.05. Filled circles show observed annual AAMRs per 100,000, standardized to the 2000 US standard population. Solid segments show the fitted joinpoint regression (NCI Joinpoint Regression Program version 6.0.1; log-linear model; uncorrelated errors; grid search; Monte Carlo permutation test with 4,499 permutations; α = 0.05). Fitted values were computed as exp(β₀ + β₁ × year) using segment-specific parameters from the Joinpoint model estimates. Parametric 95% CIs are reported for all APC and AAPC estimates. Northeast: joinpoint 2008 (95% CI, 2006-2018); segment 1 (1999-2008) APC -4.63% (95% CI, -6.87 to -2.33), p < 0.001; segment 2 (2008-2024) APC +0.93% (95% CI, -0.06 to +1.93), p = 0.065, not significant; AAPC -1.11% (95% CI, -2.09 to -0.11), p = 0.030. Midwest: joinpoint 2013 (95% CI, 2008-2016); segment 1 APC -2.49% (95% CI, -3.78 to -1.19), p < 0.001; segment 2 APC +2.41% (95% CI, +0.53 to +4.32), p = 0.014; AAPC -0.36% (95% CI, -1.39 to +0.68), p = 0.491. South: joinpoint 2014 (95% CI, 2011-2017); segment 1 APC -3.25% (95% CI, -4.47 to -2.01), p < 0.001; segment 2 APC +3.73% (95% CI, +1.43 to +6.09), p = 0.003; AAPC -0.51% (95% CI, -1.61 to +0.60), p = 0.363. West: joinpoint 2014 (95% CI, 2011-2018); segment 1 APC -3.27% (95% CI, -4.27 to -2.27), p < 0.001; segment 2 APC +2.92% (95% CI, +1.17 to +4.70), p = 0.002; AAPC -0.84% (95% CI, -1.70 to +0.03), p = 0.057. The Northeast was the only region whose second segment did not reach significance and the only region with a significantly negative AAPC. Line color denotes successive trend segments defined by the joinpoint(s): blue, the first segment; red, the second segment. AAMR, age-adjusted mortality rate; APC, annual percent change; AAPC, average annual percent change; PKD, polycystic kidney disease

The four census regions differed in joinpoint timing and in whether the second segment reached significance (Figure 4, Table 2). In the Northeast, a joinpoint was identified at 2008, with a segment-1 decline of -4.63% per year (95% CI, -6.87 to -2.33; p < 0.001) and a segment-2 change of +0.93% per year (95% CI, -0.06 to +1.93; p = 0.065) that was not significant. The Northeast AAPC was -1.11% (95% CI, -2.09 to -0.11; p = 0.030) and was significant. The Midwest showed a joinpoint in 2013 (segment 1, -2.49%; 95% CI, -3.78 to -1.19; p < 0.001; segment 2, +2.41%; 95% CI, +0.53 to +4.32; p = 0.014), with an AAPC of -0.36% (95% CI, -1.39 to +0.68; p = 0.491). The South showed a joinpoint at 2014 (segment 1, -3.25%; 95% CI, -4.47 to -2.01; p < 0.001; segment 2, +3.73%; 95% CI, +1.43 to +6.09; p = 0.003), with an AAPC of -0.51% (95% CI, -1.61 to +0.60; p = 0.363). The West showed a joinpoint at 2014 (segment 1, -3.27%; 95% CI, -4.27 to -2.27; p < 0.001; segment 2, +2.92%; 95% CI, +1.17 to +4.70; p = 0.002), with an AAPC of -0.84% (95% CI, -1.70 to +0.03; p = 0.057). Joinpoint years across regions ranged from 2008 to 2014.

Comorbidity co-mention: modeled trends

**Figure 5:**
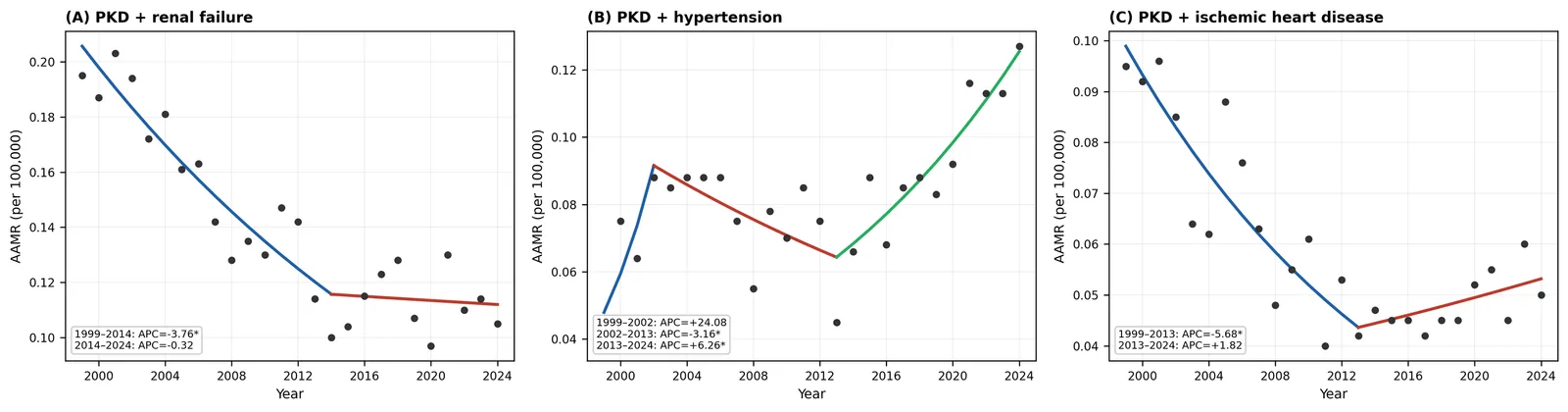
Age-adjusted co-mention rates for PKD-related deaths with selected comorbidities, US, 1999-2024: (A) Renal failure (N17-N19). (B) Hypertension (I10-I15). (C) Ischemic heart disease (I20-I25). * APC or AAPC significantly different from zero at α = 0.05. Filled circles show observed AAMRs per 100,000, standardized to the 2000 US standard population. Solid segments show the fitted joinpoint regression (Joinpoint Regression version 6.0.1; log-linear model; uncorrelated errors; grid search; Monte Carlo permutation test with 4,499 permutations; α = 0.05). Fitted values were computed as exp(β₀ + β₁ × year) using segment-specific parameters from the Joinpoint model estimates. Parametric 95% CIs are reported for all APC and AAPC estimates. Rates are age-adjusted co-mention rates, defined as deaths carrying both a qualifying PKD code and the listed comorbidity code on the same certificate. Renal failure: joinpoint 2014 (95% CI, 2007-2018); segment 1 (1999-2014) APC -3.76% (95% CI, -4.76 to -2.75), p < 0.001; segment 2 (2014-2024) APC -0.32% (95% CI, -2.42 to +1.82), p = 0.754, not significant; AAPC -2.40% (95% CI, -3.37 to -1.42), p < 0.001. Hypertension was fit with two joinpoints, at 2002 (95% CI, 2001-2008) and 2013 (95% CI, 2009-2018), yielding three segments: segment 1 (1999-2002) APC +24.08% (95% CI, -4.52 to +61.25), p = 0.101, not significant and estimated across only four observations; segment 2 (2002-2013) APC -3.16% (95% CI, -6.12 to -0.10), p = 0.044; segment 3 (2013-2024) APC +6.26% (95% CI, +3.89 to +8.68), p < 0.001; AAPC +3.92% (95% CI, +0.52 to +7.44), p = 0.023. Ischemic heart disease: joinpoint 2013 (95% CI, 2010-2018); segment 1 (1999-2013) APC -5.68% (95% CI, -7.32 to -4.02), p < 0.001; segment 2 (2013-2024) APC +1.82% (95% CI, -0.86 to +4.57), p = 0.175, not significant; AAPC -2.45% (95% CI, -3.85 to -1.04), p < 0.001. Line color denotes successive trend segments defined by the joinpoint(s): blue, the first segment; red, the second segment; green, the third segment. APC, annual percent change; AAPC, average annual percent change; PKD, polycystic kidney disease

Three co-mention series were modeled as age-adjusted rates (Figure 5, Table 2). The renal-failure co-mention rate showed a joinpoint at 2014, declining at -3.76% per year (95% CI, -4.76 to -2.75; p < 0.001) from 1999 to 2014 and changing by -0.32% per year (95% CI, -2.42 to +1.82; p = 0.754) thereafter, with an AAPC of -2.40% (95% CI, -3.37 to -1.42; p < 0.001). The hypertension co-mention rate was fit with two joinpoints, at 2002 and 2013, yielding three segments: +24.08% per year from 1999 to 2002 (95% CI, -4.52 to +61.25; p = 0.101), which was not significant and was estimated across only four observations with a correspondingly wide interval; -3.16% per year from 2002 to 2013 (95% CI, -6.12 to -0.10; p = 0.044); and +6.26% per year from 2013 to 2024 (95% CI, +3.89 to +8.68; p < 0.001). The hypertension AAPC was +3.92% (95% CI, +0.52 to +7.44; p = 0.023) and was significant. The ischemic heart disease co-mention rate showed a joinpoint at 2013, declining at -5.68% per year (95% CI, -7.32 to -4.02; p < 0.001) from 1999 to 2013 and changing by +1.82% per year (95% CI, -0.86 to +4.57; p = 0.175) thereafter, with an AAPC of -2.45% (95% CI, -3.85 to -1.04; p < 0.001).

Comorbidity co-mention: descriptive profile

Across the full period, the most frequently co-listed condition on PKD-related death certificates was renal failure, present on 8,260 certificates (48.2% of the 17,141 deaths), followed by hypertension (5,172; 30.2%), ischemic heart disease (3,795; 22.1%), heart failure (1,728; 10.1%), cerebrovascular disease (1,615; 9.4%), sepsis (1,534; 8.9%), cystic liver disease (368; 2.1%), and subarachnoid hemorrhage (293; 1.7%) (Table 3). Subarachnoid hemorrhage (I60) was queried as a nested subset of the cerebrovascular category (I60-I69) and is therefore included within that count rather than being mutually exclusive of it. Subarachnoid hemorrhage was too infrequent to support a joinpoint model. Because a single certificate may list more than one co-occurring condition, these categories do not sum to the overall total.

**Table 3:** Co-occurring causes of death listed with PKD (ICD-10 Q61.2 and Q61.3) on US death certificates, 1999-2024. ᵃ Subarachnoid hemorrhage (I60) was queried as a nested subset of the cerebrovascular disease category (I60-I69) rather than as a mutually exclusive group; the two rows therefore overlap. Each row reports deaths carrying a qualifying PKD code and the listed co-occurring cause on the same death certificate, in any position, extracted from the CDC WONDER Multiple Cause of Death files. Conditions are listed in descending order of frequency. Percentages are of the overall 17,141 PKD-related deaths. Rows do not sum to 17,141 because a single certificate may list more than one co-occurring condition, and individual decedents may therefore be represented in multiple rows. Subarachnoid hemorrhage (I60) was queried as a nested subset of the cerebrovascular disease category (I60-I69) rather than as a mutually exclusive group; those two rows therefore overlap. Subarachnoid hemorrhage was too infrequent to support a joinpoint model. Renal failure, hypertension, and ischemic heart disease were additionally modeled as age-adjusted co-mention rates and are reported in Table 2 and Figure 5. CDC WONDER, Centers for Disease Control and Prevention Wide-ranging Online Data for Epidemiologic Research; ICD-10, International Classification of Diseases, Tenth Revision; PKD, polycystic kidney disease

Co-occurring condition	ICD-10 code(s)	Deaths co-listing, N (% of 17,141)	Deaths 1999-2020	Deaths 2021-2024	Modeled by joinpoint
Renal failure	N17-N19	8,260 (48.2)	6,962	1,298	Yes
Hypertension	I10-I15	5,172 (30.2)	3,876	1,296	Yes
Ischemic heart disease	I20-I25	3,795 (22.1)	3,102	693	Yes
Heart failure	I50	1,728 (10.1)	1,316	412	No
Cerebrovascular disease	I60-I69	1,615 (9.4)	1,311	304	No
Sepsis	A40-A41	1,534 (8.9)	1,229	305	No
Cystic liver disease	Q44.6	368 (2.1)	291	77	No
Subarachnoid hemorrhageᵃ	I60	293 (1.7)	242	51	No

Underlying-cause composition

PKD (Q61.2 or Q61.3) was itself the underlying cause of death in 8,782 deaths, 51.2% of the 17,141 in the cohort. By ICD-10 chapter of the underlying cause, the largest proportion was attributed to congenital malformations, deformations, and chromosomal abnormalities, the chapter in which Q61 is classified (8,884 of 16,669 classified deaths, 53.3%), followed by diseases of the circulatory system (3,713, 22.3%), neoplasms (1,461, 8.8%), external causes (617, 3.7%), diseases of the digestive system (517, 3.1%), and diseases of the respiratory system (442, 2.7%). The small difference between the chapter figure (53.3%) and the Q61-specific figure (51.2%) reflects the few deaths whose underlying cause was a congenital anomaly other than PKD.

State-level variation

State-level deaths and age-adjusted rates are reported in Table 4. Across the 51 jurisdictions (50 states and the District of Columbia), the highest combined death counts occurred in California (2,130), Florida (1,164), Texas (1,131), New York (941), and Ohio (802). Period-specific age-adjusted rates varied widely across states in both the 1999-2020 and 2021-2024 windows. A single combined 1999-2024 AAMR was not computed because the two sub-period files use different population-estimation bases. Jurisdictions with fewer than 10 deaths in a period were suppressed, and combined state totals summed to 17,125 of the 17,141 national total, the difference reflecting these suppressed state-period cells. Because state-level rates in low-population jurisdictions rest on small annual counts, point estimates are subject to wide CIs.

**Table 4:** PKD-related deaths (ICD-10 Q61.2 and Q61.3) and AAMRs by US state, 1999-2020 and 2021-2024, with combined totals. AAMRs are per 100,000 population, standardized to the 2000 US standard population by the direct method, and are reported separately for each sub-period as provided by CDC WONDER. A single combined 1999-2024 age-adjusted rate cannot be computed from the two sub-period files because they use different population-estimation bases; combined deaths and the percentage of the national total are therefore reported instead, with percentages calculated against the 17,141 national total. Jurisdictions with fewer than 10 deaths in a period were suppressed for that period and appear as a hyphen (-). Combined state totals sum to 17,125, and the 16-death difference from the national total reflects these suppressed state-period cells. States are ranked by combined total deaths. Rates for low-population jurisdictions rest on small annual counts and carry wide CIs; point estimates for these states should not be over-interpreted. AAMR, age-adjusted mortality rate; CDC WONDER, Centers for Disease Control and Prevention Wide-ranging Online Data for Epidemiologic Research; PKD, polycystic kidney disease

State	Deaths 1999-2020	AAMR 1999-2020 (95% CI)	Deaths 2021-2024	AAMR 2021-2024 (95% CI)	Total deaths 1999-2024	% of US total
California	1774	0.333 (0.317-0.349)	356	0.300 (0.269-0.334)	2130	12.4
Florida	923	0.264 (0.246-0.282)	241	0.265 (0.229-0.307)	1164	6.8
Texas	883	0.272 (0.254-0.291)	248	0.298 (0.260-0.340)	1131	6.6
New York	778	0.251 (0.233-0.269)	163	0.263 (0.227-0.305)	941	5.5
Ohio	665	0.360 (0.332-0.389)	137	0.380 (0.321-0.450)	802	4.7
Pennsylvania	595	0.265 (0.243-0.287)	128	0.279 (0.231-0.335)	723	4.2
Washington	449	0.438 (0.396-0.479)	132	0.569 (0.477-0.675)	581	3.4
Michigan	482	0.316 (0.287-0.345)	98	0.284 (0.229-0.352)	580	3.4
Illinois	402	0.212 (0.190-0.234)	67	0.163 (0.125-0.210)	469	2.7
Minnesota	328	0.396 (0.352-0.441)	106	0.569 (0.464-0.694)	434	2.5
Wisconsin	333	0.373 (0.333-0.414)	97	0.468 (0.377-0.579)	430	2.5
North Carolina	324	0.238 (0.211-0.265)	93	0.270 (0.215-0.336)	417	2.4
Tennessee	340	0.341 (0.304-0.378)	64	0.293 (0.223-0.380)	404	2.4
Arizona	294	0.303 (0.266-0.339)	80	0.328 (0.257-0.416)	374	2.2
Oregon	287	0.457 (0.403-0.511)	81	0.563 (0.444-0.707)	368	2.1
Colorado	276	0.392 (0.344-0.440)	91	0.489 (0.387-0.612)	367	2.1
New Jersey	292	0.184 (0.162-0.206)	59	0.218 (0.169-0.278)	351	2
Indiana	284	0.303 (0.267-0.339)	65	0.306 (0.235-0.396)	349	2
Missouri	269	0.289 (0.253-0.324)	56	0.269 (0.200-0.357)	325	1.9
Maryland	251	0.292 (0.255-0.329)	68	0.333 (0.257-0.427)	319	1.9
Virginia	245	0.212 (0.185-0.240)	72	0.265 (0.208-0.336)	317	1.8
Massachusetts	244	0.232 (0.202-0.262)	66	0.292 (0.229-0.372)	310	1.8
Georgia	245	0.193 (0.167-0.219)	61	0.180 (0.134-0.237)	306	1.8
Iowa	231	0.436 (0.378-0.494)	61	0.591 (0.451-0.769)	292	1.7
Oklahoma	222	0.402 (0.348-0.455)	57	0.472 (0.353-0.621)	279	1.6
South Carolina	175	0.244 (0.207-0.282)	64	0.381 (0.294-0.491)	239	1.4
Kentucky	167	0.243 (0.205-0.281)	49	0.324 (0.234-0.442)	216	1.3
Alabama	159	0.198 (0.165-0.232)	34	0.213 (0.143-0.308)	193	1.1
Kansas	148	0.344 (0.288-0.400)	45	0.497 (0.365-0.669)	193	1.1
Connecticut	134	0.217 (0.179-0.255)	26	0.241 (0.163-0.351)	160	0.9
Louisiana	118	0.170 (0.138-0.201)	27	0.198 (0.129-0.294)	145	0.8
Nebraska	117	0.397 (0.325-0.470)	19	0.311 (0.182-0.507)	136	0.8
Arkansas	118	0.274 (0.224-0.325)	13	0.135 (0.065-0.252)	131	0.8
Utah	101	0.344 (0.276-0.412)	30	0.372 (0.250-0.537)	131	0.8
West Virginia	104	0.306 (0.246-0.366)	27	0.391 (0.243-0.615)	131	0.8
New Mexico	95	0.306 (0.246-0.376)	31	0.474 (0.318-0.689)	126	0.7
Nevada	78	0.193 (0.151-0.244)	43	0.440 (0.317-0.601)	121	0.7
Rhode Island	85	0.454 (0.362-0.563)	31	0.816 (0.551-1.190)	116	0.7
Mississippi	93	0.199 (0.159-0.246)	17	0.162 (0.083-0.291)	110	0.6
New Hampshire	86	0.396 (0.315-0.491)	20	0.388 (0.235-0.630)	106	0.6
Idaho	79	0.352 (0.277-0.441)	23	0.390 (0.241-0.606)	102	0.6
Hawaii	77	0.371 (0.291-0.465)	16	0.319 (0.183-0.540)	93	0.5
Maine	70	0.300 (0.230-0.385)	16	0.263 (0.151-0.464)	86	0.5
South Dakota	68	0.502 (0.388-0.639)	11	0.363 (0.175-0.700)	79	0.5
Vermont	60	0.561 (0.427-0.724)	16	0.575 (0.320-1.026)	76	0.4
Montana	56	0.316 (0.237-0.412)	14	0.359 (0.194-0.638)	70	0.4
North Dakota	52	0.446 (0.332-0.586)	-	-	52	0.3
Wyoming	37	0.442 (0.309-0.612)	11	0.591 (0.284-1.126)	48	0.3
Delaware	29	0.187 (0.124-0.270)	15	0.391 (0.211-0.700)	44	0.3
Alaska	30	0.364 (0.238-0.534)	-	-	30	0.2
District of Columbia	28	0.334 (0.218-0.490)	-	-	28	0.2

Sensitivity analysis

When the case definition was restricted to Q61.2 (adult-type PKD) alone, only 1,257 deaths were captured (909 in 1999-2020 and 348 in 2021-2024), 7.3% of the 17,141 in the primary cohort; the remaining 15,884 deaths (92.7%) were identified only through the unspecified code Q61.3. The share captured by Q61.2 alone was 6.6% in 1999-2020 and 10.4% in 2021-2024. Case ascertainment, therefore, depends substantially on the inclusion of Q61.3, and an analysis confined to Q61.2 would describe a small and probably unrepresentative fraction of the burden.

For the three overlapping years available in both mortality files (2018-2020), age-adjusted rates were effectively identical between the bridged-race and single-race files (2018, 0.262 vs 0.262 per 100,000; 2019, 0.255 vs 0.255; 2020, 0.290 vs 0.290), with identical death counts (672, 654, and 757, respectively). The transition between the two population-estimation schemes therefore did not materially affect the estimated rates, and the reversal, whose joinpoint (2013) precedes the 2020-2021 file boundary by seven years, cannot be an artifact of that transition.

## Discussion

Between 1999 and 2024, we identified 17,141 deaths in US adults aged 25 years and older with PKD recorded on the death certificate. Three results merit emphasis. The AAPC across the full period was -0.69% and did not differ significantly from zero, yet this apparent stability was an artifact of averaging: mortality fell by 3.01% annually through 2013 and rose by 2.34% annually thereafter, with both segments statistically significant. Second, the reversal appeared in nearly every stratum examined, but its steepness varied considerably, and the sharpest rebound occurred among non-Hispanic Black decedents at 4.17% per year, roughly double the rate among non-Hispanic White decedents. Third, the conditions recorded alongside PKD changed character. Renal failure co-mention declined and then plateaued, while hypertension co-mention accelerated after 2013, and ischemic heart disease followed the renal pattern rather than the hypertensive one. The only prior US estimate of ADPKD-specific mortality drew on the US Renal Data System and reported 18.4 and 37.4 deaths per 1,000 patient-years in non-ESRD chronic kidney disease and ESRD cohorts over 2014-2016 [2]. Those figures describe risk within a treated patient population across three years, whereas ours describe population-based rates across 26. The two are not numerically comparable, which is why the analyses are complementary rather than redundant: national certificate data capture deaths occurring outside the treated ESRD population and permit trend estimation that a short registry window cannot support.

A nonsignificant AAPC invites the conclusion that nothing happened. Here, that conclusion would have been wrong. The observed rate fell from 0.341 per 100,000 in 1999 to 0.239 in 2013, a decline of roughly 30%, then recovered to 0.311 by 2024. Averaging a significant decline against a significant rise yields a number close to zero, which describes the arithmetic accurately and the epidemiology not at all. Where a joinpoint is present, the segment-specific APCs carry the information, and the AAPC summarizes only net displacement between endpoints. Whether the reversal reflects something real about the disease or something about how deaths are recorded cannot be settled from these data, but one observation bears on it. A change in coding practice or surveillance intensity would be expected to move all subgroups by a similar proportion. Our second-segment slopes ranged from 0.93% in the Northeast to 4.17% among non-Hispanic Black decedents. Such heterogeneity across subgroups is not what a uniform change in coding or surveillance intensity would be expected to produce and is at least as consistent with differences in underlying risk or care; the descriptive design, however, cannot distinguish these explanations, and an administrative contribution cannot be excluded.

Tolvaptan was approved in the US in April 2018 as the first therapy directed at ADPKD itself [8,9]. If its introduction had begun to alter mortality, an inflection in the years following would be expected, and none occurred. The latest of the 14 joinpoints we identified fell in 2015, three years before approval. Thirteen occurred between 2008 and 2015, and the remaining one, the first of two in the hypertension co-mention series, in 2002. Six joinpoint CIs did reach 2018, and these warrant mention. They belong to the least precisely estimated series, among them the Hispanic and Northeast intervals (both 2006 to 2018) and the renal failure interval (2007-2018). No point estimate came within three years of the approval date. We therefore report this prespecified hypothesis as unsupported, and the finding is more informative than a positive result would have been, since the reversal began well before disease-specific treatment existed and treatment cannot account for it. Two further considerations reinforce this reading. Tolvaptan slows the decline in estimated glomerular filtration rate rather than acting on mortality directly [9], and in a disease that unfolds across decades, any survival signal would emerge slowly. It would also push rates downward.

The revised Kidney Allocation System took effect on December 4, 2014 [10]. Five of our 14 joinpoints fell in that same year, in the male, female, South, West, and renal failure series, and the coincidence is close enough to warrant examination. It does not survive one. Annual observations cannot distinguish a change beginning in early 2014 from one beginning in December, so the temporal ordering is uncertain at the resolution available to us. More consequentially, the direction of the policy’s effect runs opposite to what the hypothesis requires. The revision was designed to widen access for candidates disadvantaged by the previous waiting-time calculation, and preemptive transplantation became modestly more common after implementation rather than less, rising from 9.0% to 9.8% of deceased-donor transplants with an OR of 1.10 (95% CI, 1.06 to 1.14) [18]. Patients with ADPKD are heavily represented in exactly this pathway. Registry data show them to be far more likely than other patients with kidney failure to receive a preemptive transplant, with an OR of 7.13 (95% CI, 5.74 to 8.87), and more likely to be transplanted at all [19]. A policy that improved access along the route these patients preferentially travel is a poor candidate explanation for rising mortality among them, and we report this hypothesis as unsupported as well.

Men and women inflected in the same year, 2014, and their declining segments were closely matched at 3.20% and 2.95% per year. The rebounds were not. Male mortality rose at 3.68% annually against 2.11% in women, and this asymmetry, rather than any difference in the preceding decline, produced the divergence in net change. Female mortality fell significantly across the full period (AAPC -0.96%; p = 0.009) while male mortality did not (-0.50%; p = 0.328). Male predominance itself was stable, with men accounting for 9,485 deaths (55.3%) and a pooled crude rate of 0.364 per 100,000 against 0.274 in women. Mortality also rose steeply and monotonically with age, from 0.030 per 100,000 in the 25- to 34-year stratum to 1.344 among those aged 85 and older, with the largest single group aged 65-74 years (4,191 deaths). This gradient is what would be expected of a condition in which cyst burden accumulates over decades and kidney function fails late in life, and it bears on the comorbidity findings discussed below, since a population dying predominantly in its seventh and eighth decades carries substantial competing cardiovascular risk.

The racial and ethnic differences we observed lie in the slope of the rebound rather than the level of mortality. Among race and ethnicity strata, non-Hispanic White decedents inflected earliest in 2012 and non-Hispanic Black decedents latest in 2015, and the second-segment APC was higher in the latter group (+4.17% vs +2.07%). These segment slopes were estimated in separate models and were not formally compared, so the apparent difference should be regarded as exploratory rather than a demonstrated between-group difference. Neither AAPC reached significance, so the divergence is confined to the post-inflection trajectory and should not be characterized as a difference in net change across the period. Evidence on ADPKD care describes a pattern consistent with what we found. In a cohort of 41,485 patients drawn from the US Renal Data System, Black and Hispanic patients reached kidney failure at younger mean ages than White patients (55 and 53 vs 57 years) and were substantially less likely to receive a preemptive transplant, with ORs of 0.33 and 0.50, or to be transplanted after starting dialysis, with corresponding estimates of 0.61 and 0.78 [20]. Data from a large integrated health system point the same way, with Black patients experiencing the lowest rates of transplantation overall and of preemptive transplantation specifically [21]. A steeper mortality rebound in the group with the most constrained access to definitive treatment fits this picture but does not demonstrate a connection, since certificate data contain no information on transplant status and the pathway from access to death cannot be traced here. The Hispanic series behaved unlike either group, declining more sharply than any other stratum through 2010 at 9.00% per year, showing no significant change afterward, and producing the only significantly negative AAPC among race and ethnicity strata at -3.19%. Lower rates of kidney failure among Hispanic patients with ADPKD have been reported despite earlier onset [21], which may bear on this, though the Hispanic estimate rests on the smallest annual counts in our analysis and is correspondingly fragile. One additional contributor deserves mention without overstatement. Excess mortality among patients with kidney failure during the first half of 2020 was disproportionately concentrated in non-Hispanic Black, Hispanic, and Asian patients [22]. That evidence covers 15 weeks rather than the four-year span of our second segment, so it cannot account for the rebound as a whole, and the inflection preceded 2020 by five years in any case.

Three of four census regions reproduced the national pattern of decline followed by significant rebound. The Northeast did not. Its second segment rose by 0.93% annually without reaching significance (p = 0.065), and it recorded the only significantly negative regional AAPC at -1.11%. Its inflection also came earliest, in 2008, six years before those of the South and West. The South contributed the largest number of deaths (5,583; 32.6%) and the Northeast the fewest (2,869; 16.7%). At the state level, the highest counts occurred in California, Florida, Texas, New York, and Ohio, which are among the most populous states. These counts should not be read as indicating elevated risk, and rates in smaller states rest on annual counts too sparse to interpret individually. Deaths were concentrated in large central metropolitan counties, which accounted for 27.9% of deaths during 1999 to 2020, although the absence of age-adjusted rates by urbanization in the expanded file prevents any statement about trends. Regional variation in nephrology and transplant infrastructure is a plausible contributor, and disparities in transplant access among patients with ADPKD are known to differ geographically [20], but our design cannot connect regional mortality patterns to any specific structural feature.

The most informative comorbidity result is a divergence rather than any single trend. Renal failure co-mention fell by 3.76% annually through 2014 and then flattened at -0.32% (p = 0.754), producing a significant net decline of 2.40% per year. Hypertension co-mention moved the other way, rising 6.26% annually after 2013 with a significant net increase of 3.92%. Ischemic heart disease tracked renal failure rather than hypertension, falling steeply by 5.68% per year before flattening, with a significant net decline of 2.45%. Read together, these population-based co-mention rates show hypertensive disease increasingly and uremia decreasingly recorded on PKD-related death certificates, with the recent cardiovascular signal specifically hypertensive rather than ischemic. Because these are co-mention mortality rates rather than proportions among PKD-related deaths, the pattern describes changing population-level co-occurrence rather than a directly measured change in the composition of the cohort. This is compatible with what is known about the disease. Hypertension appears early in ADPKD, frequently before measurable loss of kidney function, and is the most common and most modifiable of its cardiovascular manifestations, while cardiovascular disease remains the leading cause of death [6]. The declining renal and ischemic signals also match international registry experience. Survival on renal replacement therapy improved substantially in European ADPKD cohorts, and the improvement was attributed specifically to falling cardiovascular mortality [23]. Danish registry data recorded a decline in cardiovascular and cerebrovascular deaths between 1993 and 2008 [7]. In Australia and New Zealand, the share of deaths on renal replacement therapy attributable to cerebrovascular disease fell from 15% to 6% as five-year survival improved markedly [24]. Several explanations could produce the pattern we observe, and our data do not adjudicate among them. Longer survival on renal replacement therapy shifts the terminal event later and may change what is recorded at death. Certification practice may have evolved, with hypertension documented more consistently over time. An older prevalent population, of the kind the age distribution implies, carries more accumulated cardiovascular risk. Joinpoint regression identifies when a trend changed and by how much, not why. One caveat applies to the hypertension model itself. Its first segment covers 1999-2002, rests on four observations, and carries a CI from -4.52% to +61.25%. We report it for completeness and place no interpretive weight on it.

Subarachnoid hemorrhage appeared on 293 certificates, 1.7% of the cohort, too few to support a joinpoint model. We had specified in advance that we would examine whether this co-mention declined, and the analysis could not be performed. Two readings are possible. Deaths from aneurysmal rupture may genuinely be uncommon among adults dying with PKD, or such deaths may be certified without reference to the underlying cystic disease. The second appears more likely, given that ADPKD confers substantially elevated risk of intracranial hemorrhage among patients receiving dialysis [25]. The underlying-cause distribution supports a related observation. The ICD-10 chapter containing congenital malformations, deformations, and chromosomal abnormalities, in which Q61 sits, accounted for 8,884 of 16,669 classified deaths (53.3%), with circulatory disease next at 22.3%. PKD was therefore most often the underlying cause rather than a contributing one, and the corollary is that an analysis restricted to the underlying cause would have captured roughly half of these deaths and missed the remainder. Ascertainment produced a further finding. The great majority of PKD-related deaths carried only the unspecified code Q61.3, with the adult-type code Q61.2 accounting for a small minority, indicating that certifiers rarely distinguish adult-type disease when completing the certificate. Any analysis confined to Q61.2 would describe a small and probably unrepresentative fraction of the burden. Surveillance of PKD mortality therefore depends on including the unspecified code, and the cost is genetic imprecision, a trade-off that should be stated rather than assumed away.

This analysis covers every state and the District of Columbia across 26 consecutive years, with 17,141 deaths and no sampling uncertainty at the level of ascertainment. Multiple-cause ascertainment was essential rather than incidental, since 7,785 of the 16,669 classified deaths carried a non-congenital underlying cause and would have been invisible to underlying-cause tabulation. Applying an identical joinpoint specification to all 13 series allows inflection timing and magnitude to be compared directly across strata rather than inferred from separately parameterized models. Of five prespecified hypotheses, two were refuted, and one could not be tested for want of sufficient events, and reporting them as such limits the scope for retrospective narrative. Suppression, the transition between bridged-race and single-race files, and the urbanization limitation are handled explicitly, and annual rates, model parameters, and permutation sequences are tabulated in full so that the analysis can be reproduced. Reporting follows the RECORD extension of the STROBE statement. To our knowledge, no comparable national time series exists for this condition, which is both the justification for the analysis and the reason our estimates cannot be benchmarked against a directly equivalent study.

Several limitations bear on how these results should be read. Death certificates do not establish a genetic diagnosis, and Q61.3 does not separate dominant from recessive disease. Restricting to adults aged 25 years and older substantially reduces but cannot eliminate contamination by long-surviving recessive cases, which would bias toward the null for any ADPKD-specific signal. We have no clinical information, including genotype, kidney function, imaging, dialysis or transplant status, and treatment exposure, so the mechanisms drawn from registry and cohort literature above are contextual rather than validating. Trends in recorded mortality cannot be separated from trends in recognition and certification. Co-mention indicates that two conditions appeared on the same certificate and nothing further, and because subarachnoid hemorrhage was queried as a nested subset of the cerebrovascular category, those two descriptive rows overlap.

Data-structural limitations apply as well. The transition from bridged-race to single-race files introduces a discontinuity at the 2020-2021 boundary, which falls inside the second segment. The inflections precede that boundary by five to 12 years and cannot themselves be artifacts of it, but some contribution to the magnitude of the later segment cannot be excluded. The second segment also spans the COVID-19 pandemic, whose contribution we cannot isolate. Cell suppression removed 13 deaths from age-stratified analysis and 16 from state totals, and the Hispanic and non-Hispanic Black series rest on the smallest counts. Urbanization could be examined only for 1999 to 2020. Thirteen analyses were performed without formal correction for multiplicity, so segment estimates with p-values near 0.05 warrant caution. We did not test alternative model-selection criteria such as Hudson’s method or the Bayesian information criterion, which would be unlikely to reverse direction but could shift joinpoint placement by a year or two. The design is descriptive, and no trend change reported here can be attributed causally to any particular event.

Two decades of declining PKD-related mortality in the US gave way to a rise that began in the early 2010s and has continued. That reversal preceded the first disease-specific therapy by several years, and its timing did not support a clear temporal association with the allocation-policy change with which parts of it coincide. It was not uniform: steepest among non-Hispanic Black decedents, absent in the Northeast, and absent among Hispanic decedents, whose mortality declined significantly across the period, while women achieved a net decline that men did not. The clinical picture recorded at death shifted in parallel, away from uremia and toward hypertensive disease. Because the reversal occurs in a condition with an early and treatable cardiovascular risk factor and with documented inequities in access to transplantation, continued national surveillance and attention to blood pressure control and equitable access are warranted, though these data cannot establish that any specific intervention would alter the trajectory. What remains unresolved is whether the rise reflects a change in how often the disease occurs, longer survival producing an older population at risk, or a changing certification practice.

## Conclusions

This nationwide analysis of 26 years of death certificate data identified a reversal in PKD-related mortality among US adults in 2013, with a 14-year decline followed by an 11-year rise. The reversal preceded the approval of ADPKD-specific therapy, and its timing did not support a temporal association with the 2014 revision of kidney allocation policy. The rebound was steepest among non-Hispanic Black decedents and was not observed in the Northeast or among Hispanic decedents, and women experienced a net decline across the period while men did not. Alongside these changes, the comorbidity profile recorded on death certificates shifted from renal failure toward hypertension while ischemic heart disease co-mention declined. PKD was the underlying cause of death in 51.2% of the cohort, and case ascertainment depended substantially on the unspecified ICD-10 code. Determining which of the competing explanations for this reversal predominates will require individual-level data linking genotype, kidney function trajectory, treatment exposure, and transplant access to mortality outcomes.

## Appendices

Appendix A 

**Table 5:** Annual observed AAMR, joinpoint-fitted AAMR, and standard error for PKD-related deaths (ICD-10 Q61.2 and Q61.3) in the US, 1999-2024, by stratum. Observed AAMRs are from CDC WONDER age-adjusted rate output, standardized to the 2000 US standard population. Standard errors were derived from the CDC WONDER 95% confidence limits for each rate as the interval width divided by 3.92 and supplied to the regression program as weights. Fitted values were computed from the segment-specific parameters of the final selected joinpoint model as exp(β₀ + β₁ × year), where β₀ and β₁ are the intercept and slope reported in Appendix B. Strata comprise the overall cohort, sex, race and Hispanic origin, US census region, and the three modeled comorbidity co-mention series. AAMR, age-adjusted mortality rate; CDC WONDER, Centers for Disease Control and Prevention Wide-ranging Online Data for Epidemiologic Research; ICD-10, International Classification of Diseases, Tenth Revision; IHD, ischemic heart disease; PKD, polycystic kidney disease

Year	Overall (≥25 years)			Male			Female			NH White			NH Black			Hispanic			Northeast			Midwest			South			West			PKD + renal failure			PKD + hypertension			PKD + IHD		
	Obs	Fit	SE	Obs	Fit	SE	Obs	Fit	SE	Obs	Fit	SE	Obs	Fit	SE	Obs	Fit	SE	Obs	Fit	SE	Obs	Fit	SE	Obs	Fit	SE	Obs	Fit	SE	Obs	Fit	SE	Obs	Fit	SE	Obs	Fit	SE
1999	0.341	0.375	0.0135	0.441	0.474	0.0242	0.309	0.314	0.0186	0.353	0.368	0.0158	0.415	0.483	0.0508	0.456	0.436	0.0735	0.302	0.351	0.0298	0.383	0.391	0.0304	0.313	0.335	0.0222	0.479	0.469	0.0370	0.195	0.206	0.0107	0.038	0.048	0.0048	0.095	0.099	0.0079
2000	0.405	0.364	0.0156	0.518	0.459	0.0270	0.311	0.305	0.0181	0.364	0.358	0.0158	0.482	0.468	0.0543	0.390	0.397	0.0679	0.347	0.335	0.0311	0.418	0.381	0.0314	0.330	0.324	0.0230	0.425	0.454	0.0339	0.187	0.198	0.0099	0.075	0.059	0.0066	0.092	0.093	0.0077
2001	0.331	0.353	0.0133	0.433	0.444	0.0242	0.279	0.296	0.0166	0.331	0.348	0.0151	0.500	0.453	0.0554	0.308	0.361	0.0582	0.305	0.319	0.0286	0.325	0.371	0.0270	0.318	0.313	0.0224	0.404	0.439	0.0321	0.203	0.190	0.0105	0.064	0.074	0.0059	0.096	0.088	0.0079
2002	0.371	0.342	0.0145	0.427	0.430	0.0235	0.289	0.287	0.0166	0.368	0.338	0.0161	0.430	0.439	0.0510	0.328	0.329	0.0582	0.346	0.304	0.0309	0.342	0.362	0.0288	0.319	0.303	0.0219	0.479	0.424	0.0357	0.194	0.183	0.0102	0.088	0.091	0.0069	0.085	0.083	0.0071
2003	0.313	0.332	0.0128	0.368	0.416	0.0214	0.299	0.279	0.0176	0.317	0.329	0.0145	0.407	0.425	0.0505	0.286	0.299	0.0538	0.296	0.290	0.0291	0.356	0.353	0.0293	0.290	0.293	0.0207	0.394	0.410	0.0316	0.172	0.176	0.0094	0.085	0.089	0.0074	0.064	0.078	0.0059
2004	0.322	0.322	0.0130	0.437	0.403	0.0232	0.260	0.270	0.0163	0.322	0.320	0.0145	0.418	0.412	0.0495	0.272	0.272	0.0533	0.246	0.277	0.0268	0.387	0.344	0.0309	0.272	0.284	0.0189	0.421	0.397	0.0329	0.181	0.170	0.0094	0.088	0.086	0.0074	0.062	0.074	0.0056
2005	0.344	0.312	0.0135	0.395	0.390	0.0219	0.290	0.262	0.0171	0.330	0.311	0.0151	0.408	0.399	0.0497	0.339	0.248	0.0533	0.311	0.264	0.0288	0.341	0.336	0.0276	0.287	0.274	0.0202	0.393	0.384	0.0311	0.161	0.163	0.0089	0.088	0.083	0.0071	0.088	0.070	0.0074
2006	0.309	0.303	0.0128	0.405	0.378	0.0222	0.248	0.255	0.0153	0.307	0.302	0.0140	0.386	0.386	0.0474	0.166	0.225	0.0378	0.270	0.252	0.0263	0.310	0.327	0.0265	0.307	0.265	0.0209	0.355	0.371	0.0288	0.163	0.157	0.0089	0.088	0.080	0.0066	0.076	0.066	0.0066
2007	0.279	0.294	0.0117	0.335	0.366	0.0194	0.244	0.247	0.0153	0.290	0.294	0.0135	0.417	0.374	0.0490	0.189	0.205	0.0393	0.242	0.240	0.0260	0.339	0.319	0.0273	0.235	0.257	0.0176	0.331	0.359	0.0276	0.142	0.151	0.0084	0.075	0.078	0.0061	0.063	0.062	0.0059
2008	0.262	0.285	0.0115	0.331	0.354	0.0196	0.228	0.240	0.0158	0.268	0.286	0.0135	0.289	0.362	0.0418	0.204	0.187	0.0390	0.164	0.229	0.0209	0.291	0.311	0.0250	0.203	0.248	0.0168	0.363	0.348	0.0286	0.128	0.146	0.0079	0.055	0.075	0.0054	0.048	0.058	0.0048
2009	0.276	0.277	0.0117	0.340	0.343	0.0194	0.217	0.233	0.0143	0.278	0.278	0.0133	0.352	0.351	0.0459	0.152	0.170	0.0321	0.252	0.231	0.0253	0.289	0.303	0.0250	0.228	0.240	0.0173	0.353	0.336	0.0293	0.135	0.140	0.0084	0.078	0.073	0.0066	0.055	0.055	0.0056
2010	0.278	0.268	0.0115	0.362	0.332	0.0202	0.238	0.226	0.0153	0.278	0.270	0.0135	0.397	0.340	0.0454	0.167	0.155	0.0339	0.261	0.233	0.0268	0.310	0.296	0.0270	0.245	0.233	0.0179	0.329	0.325	0.0265	0.130	0.135	0.0079	0.070	0.071	0.0059	0.061	0.052	0.0059
2011	0.243	0.260	0.0105	0.301	0.321	0.0179	0.195	0.219	0.0122	0.250	0.262	0.0122	0.348	0.329	0.0426	0.172	0.157	0.0329	0.235	0.236	0.0235	0.298	0.289	0.0245	0.224	0.225	0.0184	0.319	0.315	0.0265	0.147	0.130	0.0082	0.085	0.069	0.0066	0.040	0.049	0.0041
2012	0.260	0.252	0.0115	0.312	0.311	0.0184	0.235	0.213	0.0156	0.269	0.255	0.0133	0.268	0.319	0.0362	0.120	0.160	0.0258	0.224	0.238	0.0258	0.268	0.281	0.0245	0.238	0.218	0.0176	0.305	0.304	0.0263	0.142	0.125	0.0079	0.075	0.066	0.0061	0.053	0.046	0.0051
2013	0.239	0.245	0.0107	0.325	0.301	0.0189	0.203	0.206	0.0143	0.237	0.260	0.0125	0.320	0.309	0.0390	0.188	0.162	0.0347	0.244	0.240	0.0253	0.245	0.274	0.0219	0.226	0.211	0.0171	0.260	0.294	0.0232	0.114	0.120	0.0077	0.045	0.064	0.0041	0.042	0.044	0.0038
2014	0.269	0.250	0.0115	0.276	0.291	0.0163	0.202	0.200	0.0122	0.265	0.266	0.0135	0.286	0.299	0.0360	0.193	0.165	0.0321	0.230	0.242	0.0240	0.295	0.281	0.0245	0.188	0.204	0.0143	0.280	0.285	0.0237	0.100	0.116	0.0066	0.066	0.068	0.0054	0.047	0.044	0.0046
2015	0.273	0.256	0.0115	0.324	0.302	0.0176	0.208	0.205	0.0140	0.283	0.271	0.0135	0.281	0.290	0.0349	0.185	0.168	0.0309	0.208	0.244	0.0232	0.291	0.288	0.0240	0.211	0.211	0.0163	0.339	0.293	0.0258	0.104	0.115	0.0069	0.088	0.073	0.0069	0.045	0.045	0.0041
2016	0.259	0.262	0.0112	0.296	0.313	0.0168	0.211	0.209	0.0140	0.282	0.277	0.0135	0.234	0.302	0.0319	0.150	0.170	0.0265	0.263	0.247	0.0258	0.263	0.295	0.0235	0.206	0.219	0.0163	0.299	0.302	0.0245	0.115	0.115	0.0077	0.068	0.077	0.0056	0.045	0.046	0.0043
2017	0.253	0.268	0.0102	0.307	0.325	0.0166	0.218	0.213	0.0130	0.304	0.283	0.0145	0.359	0.314	0.0398	0.148	0.173	0.0258	0.226	0.249	0.0230	0.332	0.302	0.0270	0.229	0.228	0.0158	0.298	0.310	0.0237	0.123	0.115	0.0071	0.085	0.082	0.0069	0.042	0.047	0.0043
2018	0.262	0.275	0.0107	0.335	0.337	0.0179	0.217	0.218	0.0133	0.272	0.288	0.0130	0.313	0.327	0.0360	0.120	0.176	0.0222	0.236	0.251	0.0235	0.300	0.309	0.0237	0.226	0.236	0.0166	0.322	0.319	0.0245	0.128	0.114	0.0074	0.088	0.087	0.0064	0.045	0.048	0.0043
2019	0.255	0.281	0.0107	0.303	0.349	0.0166	0.194	0.222	0.0117	0.272	0.294	0.0135	0.324	0.341	0.0385	0.145	0.179	0.0245	0.250	0.254	0.0237	0.290	0.317	0.0253	0.211	0.245	0.0151	0.273	0.329	0.0219	0.107	0.114	0.0071	0.083	0.093	0.0061	0.045	0.049	0.0041
2020	0.290	0.288	0.0110	0.402	0.362	0.0202	0.240	0.227	0.0143	0.290	0.301	0.0125	0.385	0.355	0.0398	0.198	0.182	0.0283	0.245	0.256	0.0253	0.391	0.324	0.0288	0.269	0.254	0.0181	0.328	0.338	0.0242	0.097	0.113	0.0061	0.092	0.098	0.0061	0.052	0.049	0.0046
2021	0.323	0.294	0.0112	0.380	0.375	0.0191	0.250	0.232	0.0143	0.328	0.307	0.0145	0.392	0.370	0.0403	0.166	0.185	0.0270	0.286	0.258	0.0258	0.340	0.332	0.0263	0.304	0.263	0.0181	0.370	0.348	0.0258	0.130	0.113	0.0074	0.116	0.104	0.0071	0.055	0.050	0.0051
2022	0.328	0.301	0.0120	0.459	0.389	0.0199	0.246	0.237	0.0138	0.340	0.313	0.0151	0.430	0.386	0.0413	0.265	0.188	0.0314	0.238	0.261	0.0242	0.387	0.340	0.0283	0.315	0.273	0.0184	0.390	0.358	0.0273	0.110	0.113	0.0071	0.113	0.111	0.0074	0.045	0.051	0.0048
2023	0.283	0.308	0.0110	0.357	0.403	0.0186	0.238	0.242	0.0133	0.305	0.320	0.0138	0.412	0.402	0.0403	0.172	0.191	0.0258	0.281	0.263	0.0242	0.302	0.348	0.0250	0.256	0.284	0.0168	0.382	0.369	0.0258	0.114	0.112	0.0069	0.113	0.118	0.0069	0.060	0.052	0.0054
2024	0.311	0.316	0.0110	0.395	0.418	0.0179	0.236	0.247	0.0133	0.318	0.326	0.0140	0.342	0.418	0.0367	0.178	0.194	0.0255	0.278	0.266	0.0247	0.318	0.357	0.0242	0.259	0.294	0.0173	0.357	0.380	0.0250	0.105	0.112	0.0066	0.127	0.125	0.0069	0.050	0.053	0.0051

Appendix B 

**Table 6:** Joinpoint regression: final selected model and segment parameters for each analytic stratum. Final selected joinpoint model for each stratum, showing the number of joinpoints, joinpoint year with 95% CI, number of observations, model parameters, residual degrees of freedom, sum of squared errors, mean squared error, and the segment-specific intercept (β₀) and slope (β₁, on the log-rate scale). Joinpoint regression was fit using the NCI Joinpoint Regression Program version 6.0.1 with a log-linear model, uncorrelated errors, and grid search, requiring a minimum of two observations between adjacent joinpoints and between any joinpoint and the series endpoints. The joinpoint year and its CI are shown on the segment beginning at that joinpoint. The permutation-test sequence that determined the number of joinpoints for each stratum is given in Appendix C. DF, degrees of freedom; IHD, ischemic heart disease; MSE, mean squared error; PKD, polycystic kidney disease; SSE, sum of squared errors

Stratum	# joinpoints	N obs	# param	Residual DF	SSE	MSE	Segment	Joinpoint (95% CI)	Intercept	Slope (log-rate/yr)
Overall (U.S., adults ≥25 years)	1	26	4	22	69.5132	3.159691	Segment 0	None	60.08798	-0.03055
							Segment 1	2013 (2010-2016)	-47.9431	0.023117
Male	1	26	4	22	62.14149	2.824613	Segment 0	None	64.19882	-0.03249
							Segment 1	2014 (2011-2017)	-74.0023	0.036132
Female	1	26	4	22	23.37803	1.062638	Segment 0	None	58.73972	-0.02996
							Segment 1	2014 (2010-2017)	-43.712	0.020906
Non-Hispanic White	1	26	4	22	30.96124	1.407329	Segment 0	None	55.39657	-0.02821
							Segment 1	2012 (2009-2014)	-42.5298	0.020459
Non-Hispanic Black	1	26	4	22	20.93406	0.951548	Segment 0	None	63.12214	-0.03194
							Segment 1	2015 (2012-2017)	-83.614	0.040881
Hispanic or Latino	1	26	4	22	28.53996	1.297271	Segment 0	None	187.7664	-0.09435
							Segment 1	2010 (2006-2018)	-34.5396	0.016255
Northeast	1	26	4	22	25.89908	1.177231	Segment 0	None	93.61724	-0.04736
							Segment 1	2008 (2006-2018)	-20.0416	0.009247
Midwest	1	26	4	22	30.48426	1.385648	Segment 0	None	49.51596	-0.02524
							Segment 1	2013 (2008-2016)	-49.2614	0.023829
South	1	26	4	22	42.49328	1.931513	Segment 0	None	64.93497	-0.03303
							Segment 1	2014 (2011-2017)	-75.4394	0.036668
West	1	26	4	22	22.06684	1.003038	Segment 0	None	65.7455	-0.03327
							Segment 1	2014 (2011-2018)	-59.2213	0.028781
PKD + renal failure	1	26	4	22	51.20029	2.327286	Segment 0	None	75.02806	-0.03832
							Segment 1	2014 (2007-2018)	4.392617	-0.00325
PKD + hypertension	2	26	6	20	67.18683	3.359341	Segment 0	None	-434.398	0.215788
							Segment 1	2002 (2001-2008)	61.80505	-0.03207
							Segment 2	2013 (2009-2018)	-124.927	0.060697
PKD + IHD	1	26	4	22	41.40234	1.881924	Segment 0	None	114.6203	-0.0585
							Segment 1	2013 (2010-2018)	-39.3983	0.018016

 Appendix C  

**Table 7:** Joinpoint regression: Monte Carlo permutation test sequence for each analytic stratum. Monte Carlo permutation test sequence used to select the optimal number of joinpoints in each stratum. Each row reports the test number, the null and alternative joinpoint counts, the number selected at that step, numerator and denominator degrees of freedom, the number of permutations, the resulting p-value, and the per-test significance threshold. Model selection followed the method of Kim and colleagues with 4,499 permutations per test and an overall significance level of α = 0.05. The final accepted model for each stratum and its segment parameters are reported in Appendix B. DF, degrees of freedom; H₀, null hypothesis; H₁, alternative hypothesis; IHD, ischemic heart disease; JP, joinpoint; PKD, polycystic kidney disease

Stratum	Test #	H0 (# JPs)	H1 (# JPs)	Selected	Num. DF	Den. DF	# permutations	p-value
Overall (U.S., adults ≥25 years)	0	0	4	4	8	16	4500	0.000222
	1	1	4	1	6	16	4500	0.327778
	2	1	3	1	4	18	4500	0.390444
	3	1	2	1	2	20	4500	0.883556
Male	0	0	4	4	8	16	4500	0.000222
	1	1	4	1	6	16	4500	0.432222
	2	1	3	1	4	18	4500	0.251778
	3	1	2	1	2	20	4500	0.196889
Female	0	0	4	4	8	16	4500	0.000222
	1	1	4	1	6	16	4500	0.577556
	2	1	3	1	4	18	4500	0.612444
	3	1	2	1	2	20	4500	0.822889
Non-Hispanic White	0	0	4	4	8	16	4500	0.000222
	1	1	4	1	6	16	4500	0.156444
	2	1	3	1	4	18	4500	0.606667
	3	1	2	1	2	20	4500	0.664667
Non-Hispanic Black	0	0	4	4	8	16	4500	0.001111
	1	1	4	1	6	16	4500	0.234889
	2	1	3	1	4	18	4500	0.102667
	3	1	2	1	2	20	4500	0.061333
Hispanic or Latino	0	0	4	4	8	16	4500	0.000444
	1	1	4	1	6	16	4500	0.070222
	2	1	3	1	4	18	4500	0.136444
	3	1	2	1	2	20	4500	0.359778
Northeast	0	0	4	0	8	16	4500	0.022
	1	0	3	0	6	18	4500	0.013778
	2	0	2	2	4	20	4500	0.003333
	3	1	2	1	2	20	4500	0.327333
Midwest	0	0	4	4	8	16	4500	0.004889
	1	1	4	1	6	16	4500	0.264444
	2	1	3	1	4	18	4500	0.245111
	3	1	2	1	2	20	4500	0.085111
South	0	0	4	4	8	16	4500	0.000222
	1	1	4	1	6	16	4500	0.081556
	2	1	3	1	4	18	4500	0.042222
	3	1	2	1	2	20	4500	0.026667
West	0	0	4	4	8	16	4500	0.000444
	1	1	4	1	6	16	4500	0.237111
	2	1	3	1	4	18	4500	0.210444
	3	1	2	1	2	20	4500	0.667778
PKD + renal failure	0	0	4	0	8	16	4500	0.041333
	1	0	3	0	6	18	4500	0.075333
	2	0	2	0	4	20	4500	0.043556
	3	0	1	1	2	22	4500	0.011556
PKD + hypertension	0	0	4	0	8	16	4500	0.012889
	1	0	3	3	6	18	4500	0.004
	2	1	3	1	4	18	4500	0.063556
	3	1	2	2	2	20	4500	0.011778
PKD + IHD	0	0	4	4	8	16	4500	0.004444
	1	1	4	1	6	16	4500	0.805111
	2	1	3	1	4	18	4500	0.9100000
	3	1	2	1	2	20	4500	0.9360000

Appendix D  

**Table 8:** Annual PKD-related deaths (ICD-10 Q61.2 and Q61.3) in the US, 1999-2024, by demographic, geographic, age, and comorbidity stratum. Counts extracted from the CDC WONDER Multiple Cause of Death files (bridged-race file 1999–2020; single-race file 2021-2024) for adults aged 25 years and older. Annual cells containing fewer than 10 deaths are suppressed in CDC WONDER output and appear as an em-dash per NCHS confidentiality rules. Column totals are sums of visible annual values and may therefore fall below the true stratum total where suppression occurred; the overall column is the sum of the seven age bands and totals 17,128 against the unsuppressed national total of 17,141. Race categories are non-Hispanic; Hispanic origin is tabulated separately. Comorbidity columns report deaths carrying both a qualifying PKD code and the listed condition on the same certificate. CDC WONDER, Centers for Disease Control and Prevention Wide-ranging Online Data for Epidemiologic Research; ICD-10, International Classification of Diseases, Tenth Revision; NH, non-Hispanic; NCHS, National Center for Health Statistics; PKD, polycystic kidney disease

Year	Overall (≥25 years)	Male	Female	NH White	NH Black	Hispanic	Northeast	Midwest	South	West	25-34	35-44	45-54	55-64	65-74	75-84	85+	Renal failure	Hypertension	IHD	Heart failure	Cerebrovascular	SAH	Sepsis	Cystic liver
1999	643	348	295	511	73	47	106	163	204	170	10	41	91	129	173	143	56	358	96	170	64	91	15	54	-
2000	682	376	306	535	88	43	128	179	211	164	21	47	106	135	157	156	60	381	165	161	60	79	23	73	19
2001	621	334	293	491	90	34	115	147	204	161	-	47	93	146	134	151	50	380	159	162	47	53	-	67	16
2002	670	352	318	535	79	39	129	143	217	181	13	37	103	136	160	161	60	375	175	158	52	71	-	69	14
2003	620	316	304	487	73	39	108	150	201	161	12	37	101	137	138	137	58	355	149	145	51	62	12	63	20
2004	640	365	275	511	79	35	94	165	212	169	11	35	96	128	147	153	70	377	159	151	68	79	24	65	15
2005	653	348	305	509	76	50	125	154	209	165	10	38	108	141	131	147	78	339	181	156	56	93	21	71	18
2006	617	349	275	501	75	27	106	143	216	159	-	43	106	125	126	151	66	349	191	142	47	56	-	63	11
2007	591	316	275	458	82	32	95	156	188	152	10	46	101	120	108	138	68	298	178	132	44	56	16	47	13
2008	539	306	233	425	54	38	73	137	161	168	10	37	71	133	124	111	53	261	150	124	44	53	10	48	20
2009	585	326	259	466	68	30	103	137	184	161	10	38	72	142	132	120	71	290	165	121	58	50	13	47	-
2010	607	337	270	463	88	36	103	141	201	162	19	36	89	144	134	140	45	281	180	139	51	59	15	40	14
2011	581	311	270	446	78	34	103	157	170	151	11	20	84	126	154	105	81	337	173	121	47	47	10	48	14
2012	571	310	261	455	63	31	90	134	196	151	11	27	75	127	142	119	70	336	164	124	51	47	-	60	12
2013	563	317	246	425	78	38	103	132	191	137	19	27	61	115	143	126	72	254	160	141	55	43	10	47	16
2014	605	315	290	467	74	46	101	158	191	155	12	23	75	148	158	109	80	256	184	126	56	43	10	56	13
2015	627	359	268	478	74	45	96	154	190	187	10	33	69	159	153	125	78	273	201	134	59	45	10	43	-
2016	602	336	266	478	63	38	120	139	187	156	10	29	73	141	129	138	82	268	173	124	58	51	16	47	11
2017	667	367	300	504	95	40	107	170	219	171	17	45	75	140	185	125	80	307	182	134	71	55	13	47	17
2018	672	366	306	515	86	39	111	168	203	190	12	36	76	135	182	143	88	311	208	137	87	58	10	59	15
2019	654	368	286	503	85	43	119	152	216	167	13	25	66	138	191	135	86	276	216	142	91	58	14	55	15
2020	757	430	327	566	101	57	109	200	253	195	19	54	90	154	183	173	84	300	267	158	99	62	-	60	18
2021	819	468	351	617	101	63	128	181	298	212	14	35	103	174	223	168	102	335	317	170	92	68	10	80	17
2022	903	531	372	664	116	85	127	223	303	250	18	48	112	181	249	192	103	336	337	168	114	85	11	75	21
2023	814	479	335	591	119	59	133	183	271	227	16	47	82	179	218	170	102	313	314	179	101	68	16	71	17
2024	825	455	370	600	102	71	137	180	287	221	25	55	80	146	217	201	101	314	328	176	105	83	14	79	22
Total	17128	9485	7656	13201	2160	1139	2869	4146	5583	4543	333	986	2258	3679	4191	3737	1944	8260	5172	3795	1728	1615	293	1534	368
